# Single‐cell RNA landscape of cell fate decision of renal proximal tubular epithelial cells and immune‐microenvironment in kidney fibrosis

**DOI:** 10.1002/ctm2.1010

**Published:** 2022-09-09

**Authors:** Minyan Zhu, Zhenhua Zhang, Zhejun Chen, Yao Xu, Jiajin Wu, Xiajing Che, Liang Ying, Xinghua Shao, Lumin Tang, Wenyan Zhou, Minfang Zhang, Ming Zhang, Shan Mou

**Affiliations:** ^1^ Department of Nephrology Molecular Cell Lab for Kidney Disease Renji Hospital School of Medicine Shanghai Jiao Tong University Shanghai China; ^2^ School of Pharmaceutical Sciences Sun Yat‐sen University Guangzhou Guangdong China; ^3^ Department of Urology Renji Hospital School of Medicine Shanghai Jiao Tong University Shanghai China; ^4^ Transplantation Center of Renji Hospital School of Medicine Shanghai Jiao Tong University Shanghai China

Dear Editor,

This study found that proximal tubular epithelial cells (PTECs), the main population of cells in the kidneys, exhibited transcriptional heterogeneity during interstitial fibrosis and tubular atrophy (IFTA), which contributes to the complex metabolic and immuno‐microenvironment. The activated transcription factor (TF) NR1H4 regulated the differentiation and maladaptive repair of PTECs by targeting the phenylalanine hydroxylase (PAH) gene.

IFTA is a shared pathological change that determines the transition and progression of chronic kidney disease (CKD).^1^ The mechanism underlying the maladaptive repair of PTECs and their crosstalk with the immuno‐microenvironment remain unclear. To better understand the transcriptional dynamics and cell composition in IFTA, we characterized the molecular profiles of 21 466 cells from kidney biopsy samples of one donor, two mild IFTA (one membranous nephropathy and one lupus nephritis) and two moderate‐to‐severe IFTA (one IgA nephropathy and one chronic tubulointerstitial nephritis) through scRNA‐sequencing. Clinical information, annotated cell clusters and methods can be found in Supplementary Methods, Tables S1 and S2, Figures 1A,B and S1–S8.

**FIGURE 1:**
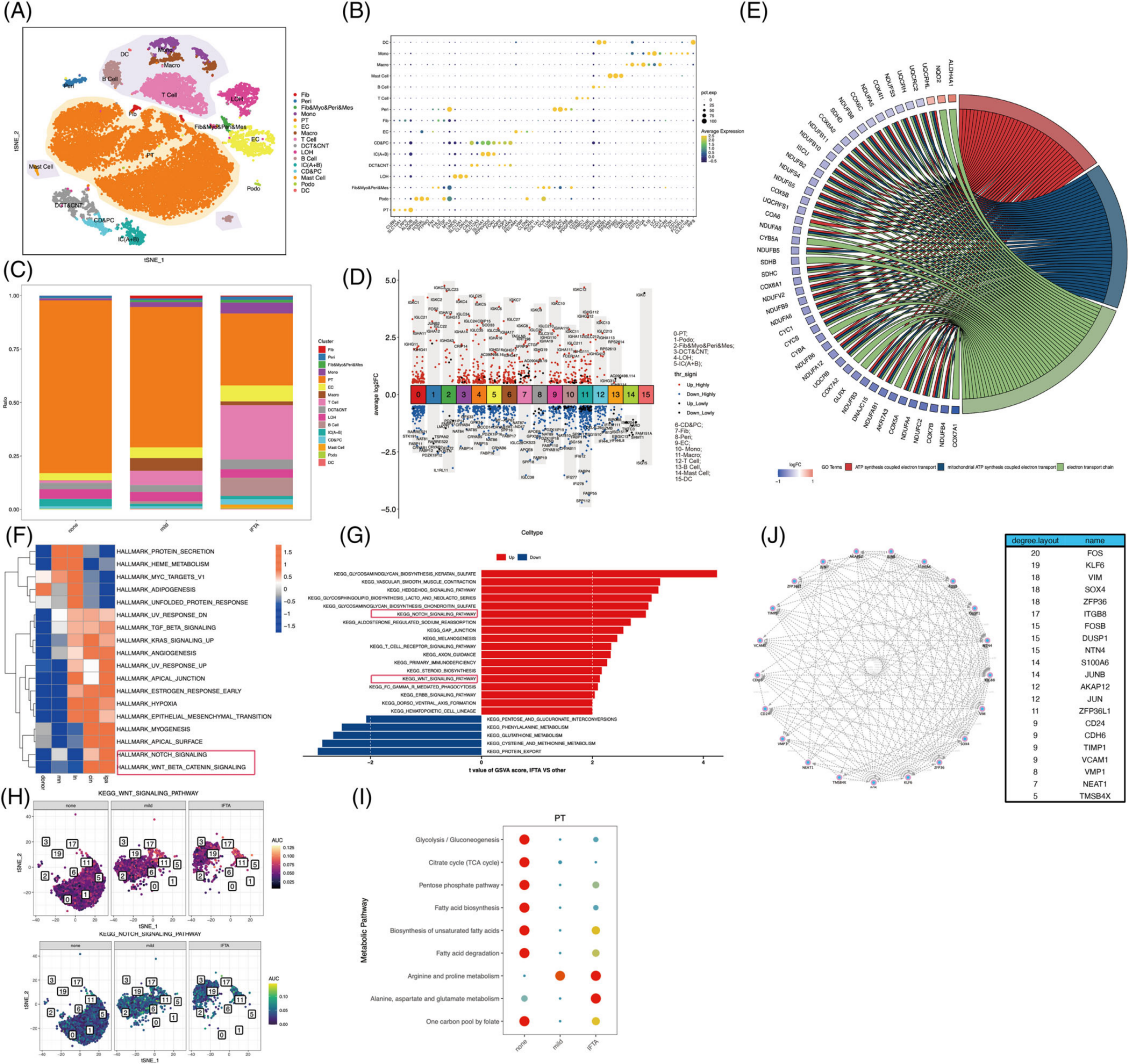
Transcriptomic analysis of kidney interstitial fibrosis and tubular atrophy (IFTA) at a single‐cell level and the transcriptional heterogeneity of proximal tubular epithelial cells (PTECs). (A) Unbiased clustering of cells from all samples by *t*‐SNE; (B) a bubble plot of the expression of markers for specific populations of cells; (C) the relative cell proportion across three IFTA grades; (D) the differentially expressed genes (DEGs) among the cell population of the IFTA samples compared to others. The labelled genes were the top five upregulated or downregulated DEGs ranked by the average log2FC; (E) a circle plot showing the top three changed pathways and the corresponding top regulated genes according to the enrichment analysis based on DEGs in (D) of PTECs; (F) the results of the GSVA regarding pathway activity across different samples visualized by a heat map. The analysis was based on the Hallmark of the MSigDB database; (G) the results of the GSVA of the pathway activity in the IFTA group compared to others visualized by a bar plot. The analysis was based on KEGG of the MSigDB database; (H) AUCell was used to visualize the relatively upregulated activity of the cell differentiation‐related pathways (NOTCH and WNT‐β‐catenin) with the progression of IFTA; (I) the metabolic pathway activity score of PTECs was determined by the scMetabolism method. PTECs showed lower glucose and lipid metabolism but higher arginine and glutamate metabolism with the development of IFTA; (J) an interaction network analysis of the transcriptional heterogeneous hub genes for IFTA was performed based on WGCNA. The hub genes are listed on the right. The degree layout represents the number of interactions of the hub genes

We found that samples with IFTA had fewer PTECs, with more immune cells than that present in other samples (Figures 1C and S4). Enrichment analysis based on differentially expressed genes (DEGs) of PTECs in IFTA versus the others indicated significantly lower energy metabolism and higher mRNA catabolic processes, ribosome‐related pathways, cell apoptosis and biosynthesis of amino acids (Figures 1D‐E1 and S9). A GSVA and AUCell analysis of all genes between IFTA and the others demonstrated that the amount and activity of hedgehog, notch and WNT pathways (related to cell development in renal fibrosis^2^) were significantly elevated (Figures 1F–H and S9). Lower glucose and lipid metabolism and higher arginine and glutamate‐related metabolism were further found in the PTECs of the IFTA group by the scMetabolism and ssGSEA analysis (Figures 1I and S10a–e), indicating a possible relationship between protein metabolism and the cell differentiation of PTECs. In contrast, TCA cycle was elevated in the mast cells of the mild group, whereas fatty acid degradation and one‐carbon metabolism elevated in the T cells of the IFTA group, and pentose phosphate pathway elevated in the B cells of the IFTA group, suggesting probable changes in cell states and the metabolic microenvironment. FOS (regulating TGF‐β‐mediated signalling^3^), KLF6 (mediating branched‐chain amino acid catabolism loss in PTECs^4^), VIM and SOX4 (related to the differentiation of PTECs^5^) were the top hub genes related to IFTA grades found by WGCNA (Figures 1J and S11).

To determine whether heterogeneous PTECs contribute to different immuno‐microenvironments during the progression of IFTA besides the probable effects of metabolism, cell crosstalks were calculated by CellChat (Figures 2 and S12–16). PTECs interact with immune cells mainly by the MIF and SPP1 pathways, and the strongest interactions were with monocytes in all samples (Figures 2A,B and S12e). MIF‐(CD74 + CXCR4) took most account of MIF pathway from PTECs which were the strongest to dendritic cells (DCs) (Figures 2C and S13f). PTECs received the main signalling of PTN and MK from (myo)fibroblast‐like cells instead of immune cells (Figures 2D and S12e, S14). As IFTA progressed, the communication of PTECs with immune cells increased by activating CXCR4 in monocytes, macrophages, T cells, B cells and DC cells, and CD44 in monocytes, macrophages, T cells and mast cells with MIF (Figure 2E–G). The activation in monocytes and macrophages was the most prominent among all immune cells (Figures S15d and 16d). The hyperactivation of CXCR4 and CD44 receptors in immune cells is proinflammatory and promotes kidney injury.^6^

**FIGURE 2:**
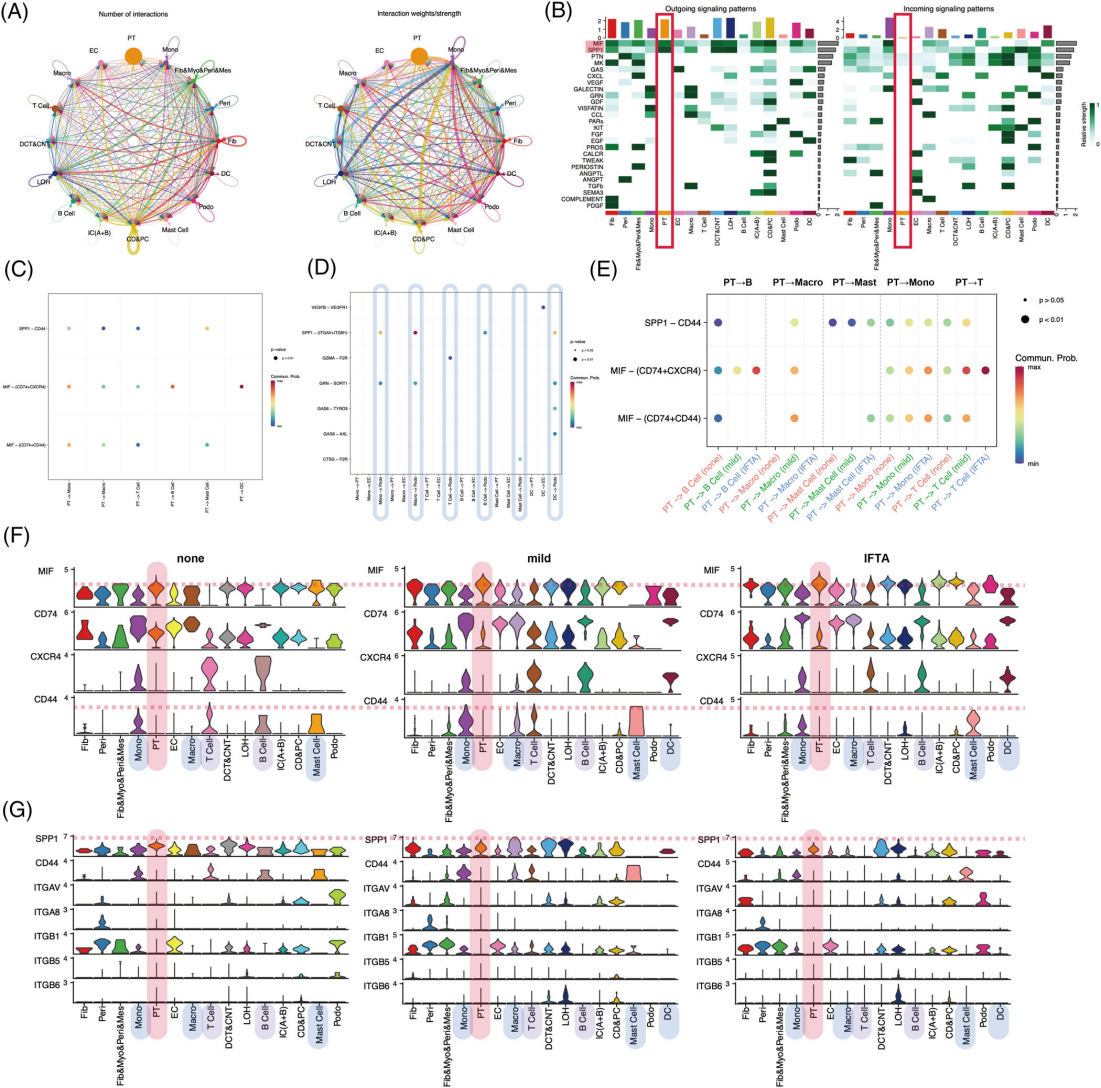
The crosstalk between proximal tubular epithelial cells (PTECs) and the infiltrated immune cells during kidney interstitial fibrosis and tubular atrophy (IFTA). (A) A circle plot showing the interactions among cell types across all samples regarding the number (left) and the weight/strength of the interactions (right); (B) a heat map summarizing the outgoing (secreting) and incoming (target) signal pathways of each cell group among all samples; bubble plots of the main signalling pathways from PTEC (C) and infiltrated immune cells (D); (E) a bubble plot summarizing the comparison of signalling pathways from PTEC to infiltrated immune cells across three IFTA grades; a violin plot showing the detailed expression of the genes related to the MIF pathway (F) and the SPP1 pathway (G) in each cell types among different IFTA grades. CD&PC, connecting ducts and principal cells; DC, dendritic cells; DCT&CNT, distal convoluted and connecting tubules; EC, endothelial cells; Fib, fibroblast; Fibro&Myofib&Peri&Mes, myofibroblast‐like cells, including fibroblasts, myofibroblasts, pericytes and mesangial cells; IC (A + B), intercalated cell types A and B of the collecting duct; LOH, loop of Henle; Macro, macrophages; Mono, monocytes; PT, proximal tubular epithelial cells; Peri, pericytes; Podo, podocytes

PHATE 3D also visualized that PTECs were the main cell cluster that underwent cell differentiation as IFTA progressed (Figures 3A and S17a). Two Seurat clusters (17, 11) of high capacity were further found to differentiate into normal cells in the none group or clusters (2, 3, 6, 19) with the highest expression of injury molecule (HAVCR1 and PDGFB) (Figures 3B–D and S17b–d) through the RNA velocity and PAGA algorithm. The pseudotime trajectory analysis was next performed using Monocle2 to determine the DEGs and pathways related (Figures 3 and S18). PTECs were differentiated into two different branches (states), where Seurat clusters 2 and 3 (most of which were from the mild or IFTA group) were the downstream of differentiation (Figures 3E,F and S18b). The cells of Seurat clusters 2 and 3 (belonging to cell state 5) showed high protein processing in the ER, fluid shear stress and proinflammatory pathways but low fatty acid degradation (Figure 3G–I).

**FIGURE 3:**
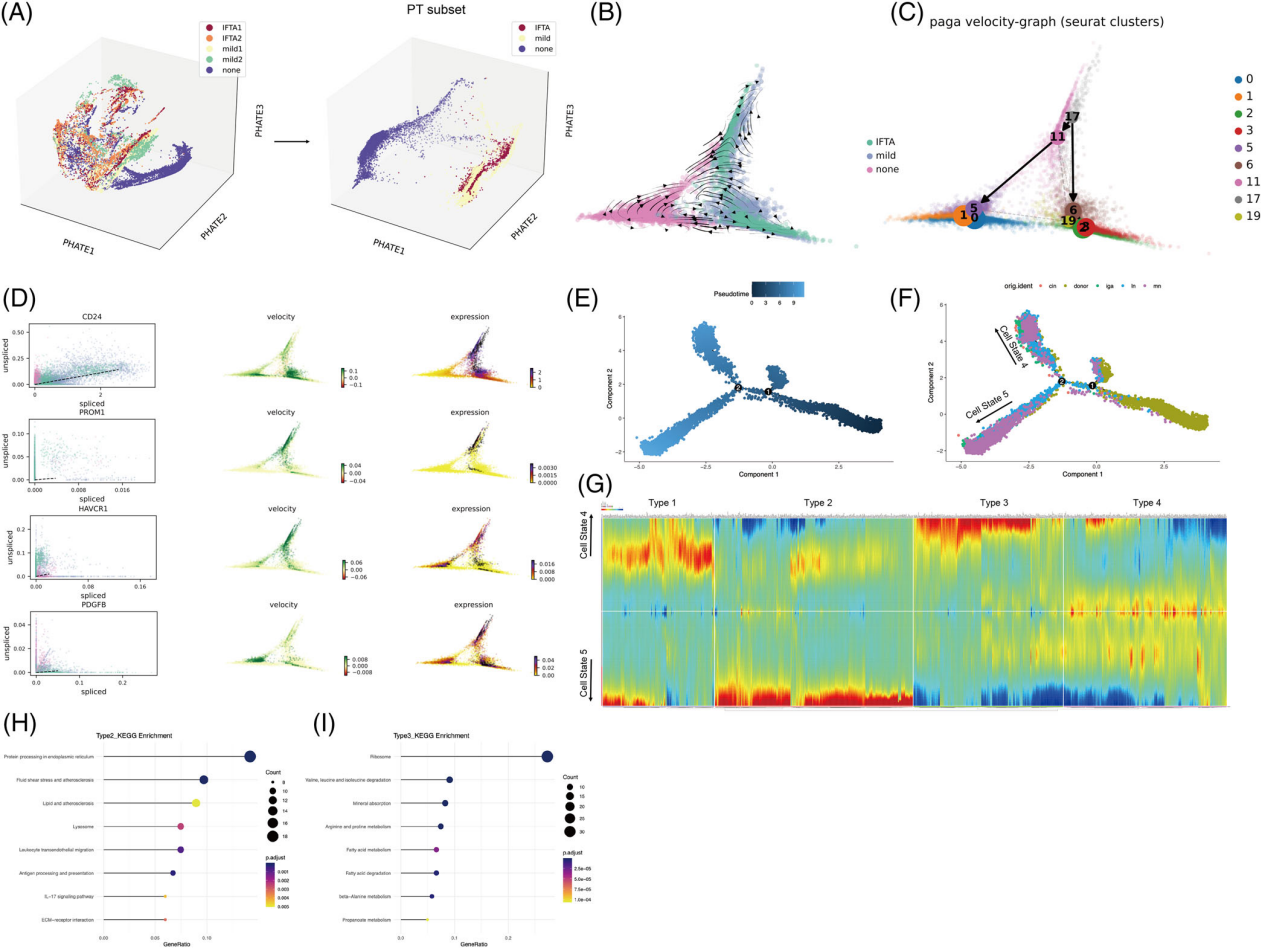
The trajectory of the analysis of proximal tubular epithelial cells (PTECs) in kidney fibrosis. (A) The 3D PHATE embedding of all cells (left) and PTECs (right) among all samples. None: donor kidney, Mild1: kidney with membranous nephropathy (MN), Mild2: kidney with lupus nephropathy (LN). Interstitial fibrosis and tubular atrophy (IFTA)1: kidney with chronic tubulointerstitial nephritis (CIN); IFTA2: kidney with IgA nephropathy (IgA). Mild: the group of mild1 and mild2, IFTA: the group of IFTA1 and IFTA2; (B) RNA velocity inference of the differentiation trajectory of PTECs along with IFTA progression; (C) the PAGA graph of the inferred differentiation trajectory of PTECs annotated with Seurat clusters for all samples; (D) the transcriptional dynamics of the interested genes CD24, RPOM1 (CD133), HAVCR1 (KIM1) and PDGFB, which are related to tubular differentiation or injury with changes in the trajectory, are shown. The estimated steady ratio of unspliced to spliced mRNA of the gene of interest is represented by the dotted line in black in the left four plots; (E) the target gene is considered to be increasing if most of the dots are above the black line; the trajectory plot was plotted using Monocle2 for showing the dynamics of the cell state with the pseudotime curve (E) and sample origin (F); (G) the differentially expressed genes (DEGs) (*p* < 10^−10^) that changed along the pseudotime trajectory from the second branch point were hierarchically clustered into four types; (H) the enrichment analysis of the genes in type 2 (increase in cell state 5 and decrease in cell state 4) based on the KEGG database; (I) the enrichment analysis of genes in type 3 (decrease in cell state 5 and increase in cell state 4) based on the KEGG database. PT, proximal tubular epithelial cells

To determine the potential TFs regulating the transcriptional heterogeneity and differentiation of PTECs, SCENIC analysis was conducted (Figures 4 and S19–28). HNF4A (579 g), NR1H4 extended (191 g), NR2F1 extended (295 g), HNF1A (358 g) and POLE4 extended (431 g) were the top five specific regulons (TF‐target gene) in PTECs, among which NR1H4 extended (191 g) and HNF4A (579 g) (that regulates adaptive repair of PTECs^7^) were the most active, as determined by the average and binary algorithm (Figures 4C and S22). Tubular NR1H4 expression was negatively correlated with eGFR (*r* = −.537, *p* = .004), whereas HNF4A positively, through analysing public RNA‐seq data^8^, ^9^ (Figure S23a,b). The PTECs highly expressing NR1H4 also secreted more proinflammatory CCL15 and C3 to immune cells and showed lower glucose and fatty acid metabolism than the PTECs lowly expressing NR1H4 (Figures 4D and S26, S27). A Venn diagram and motif enrichment analysis showed that NR1H4‐PAH (monocle2 type3 gene) had a binding motif of high confidence (details in Table S6), also predicted by the JASPAR database (Figure S24e). NR1H4 levels were high, and PAH levels were low in mice with folic acid‐induced nephropathy (FAN) and H_2_O_2_‐stimulated HK‐2 cells (Figures 4F and S28a,b). Mice in which NR1H4 was knocked out or administered the NR1H4 inhibitor *z*‐guggulsterone or Gly‐β‐MCA demonstrated less IFTA and apoptosis of PTECs with partially recovered PAH expression (Figures 4G–K and Figure S28c) in FAN. These results were similar to our previous findings on the ischaemia‐reperfusion model.^10^ The HK‐2 cells in which PAH was knocked down underwent more severe apoptosis under H_2_O_2_ modelling (Figure S28e).

**FIGURE 4:**
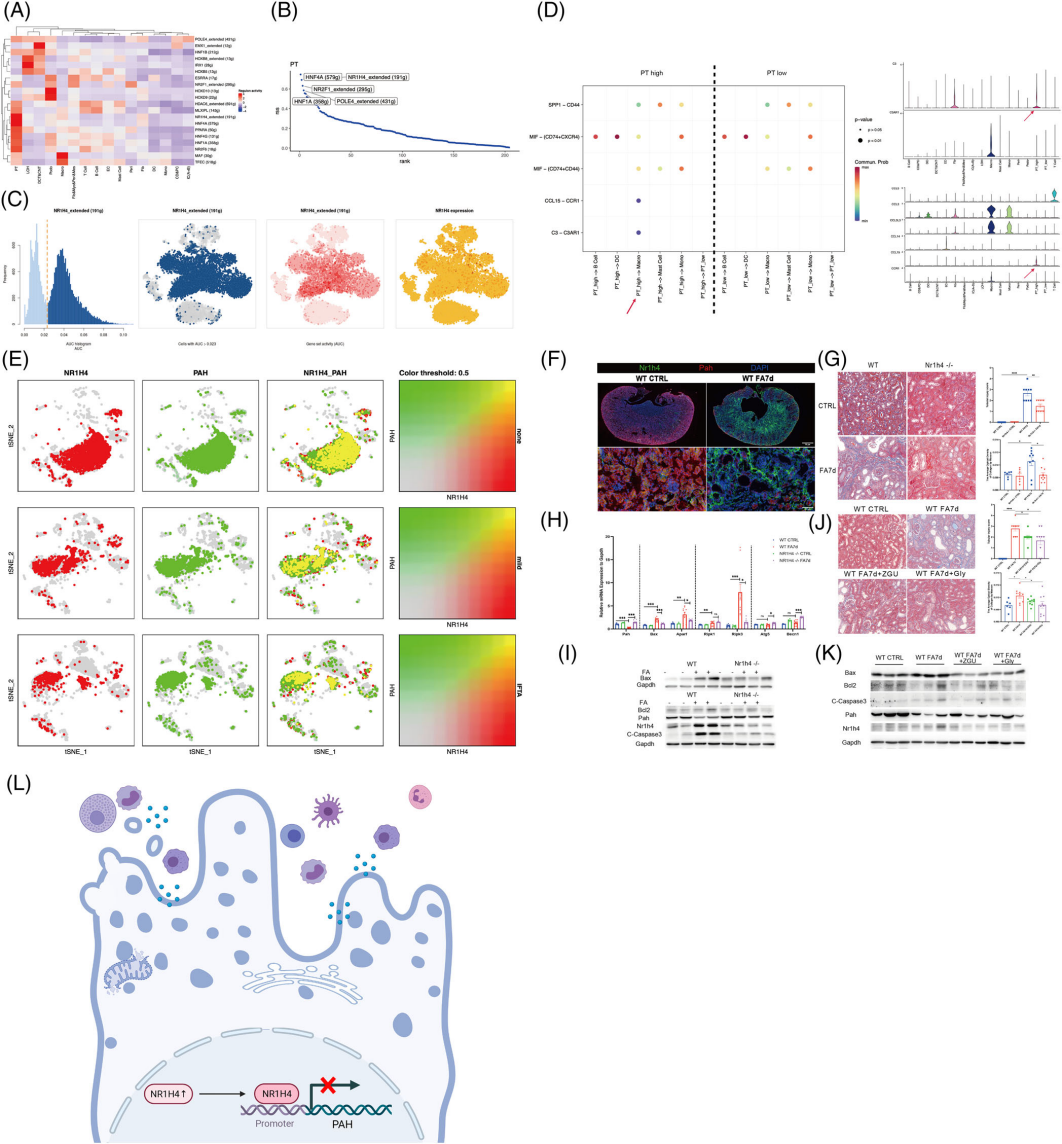
Transcription factors (TFs) and corresponding target genes specific to cell types by the SCENIC and validation of NR1H4‐phenylalanine hydroxylase (PAH) in proximal tubular epithelial cells (PTECs). (A) The regulation of TFs among different types of cells by the average algorithm; (B) the top five regulons (TFs and corresponding target gene candidates) in PTECs; (C) the activity of the NR1H4 regulons of PTECs by the average algorithm. The yellow line in the left plot represents the threshold of AUCs (.023) for the active expression of the genes in the NR1H4 regulon among PTECs. The *y*‐axis of the left plot represents the cell numbers corresponding to the AUC value on the *x*‐axis. The blue points in the middle left plot represent cells with regulon AUC > .023, whereas the grey points represent cells with regulon AUC ≤ .023. The red points in the middle right plot represent the original regulon AUC value of each cell, among which the darker red points indicate a greater regulon AUC value. The red points in the right plot represent the NR1H4 expression of each cell; (D) bubble plots of the main signalling pathway from PTECs to infiltrated immune cells among all samples (left) and detailed gene expression in the COMPLEMENT and CCL pathways, which is different among the PTECs in which NR1H4 is highly expressed and those in which NR1H4 is poorly expressed. PT high: NR1H4 highly expressed PTECs (count of NR1H4 > 0); PT low: NR1H4 poorly expressed PTECs (count of NR1H4 = 0); (E) the co‐localization and relatively higher expression of NR1H4 and PAH in PTECs among samples with different grades of interstitial fibrosis and tubular atrophy (IFTA); (F) immunofluorescence of NR1H4 (green) and PAH (red) in the mouse kidney with and without folic acid (FA) injection. PAH: phenylalanine hydroxylase; (G) Masson staining and semi‐quantification of injured tubules and deposited collagens in WT and NR1H4^−/−^mice with and without FA injection; (H) the expression of PAH, apoptosis‐related (Bax and Aparf), necroptosis‐related (Ripk1 and Ripk3) and autophagy‐related (Atg5 and Becn1) mRNAs in WT and NR1H4^−/−^mice kidney with and without FA injection; (I) the expression of NR1H4, PAH and apoptosis‐related proteins was determined by performing the Western blot assay in the WT and NR1H4^−/−^mouse kidney with and without FA injection. (J) Masson staining and semi‐quantification of injured tubules and deposited collagens were performed in WT mice administered with or without the NR1H4 inhibitor *z*‐guggulsterone (ZGU) or Gly‐β‐MCA (Gly); (K) the expression of NR1H4, PAH and apoptosis‐related proteins was determined by performing the Western blot assay in WT mice gavaged with or without the NR1H4 inhibitor ZGU or Gly; (L) graphic abstract. PT, proximal tubular epithelial cells

In summary, we found that heterogeneous PTECs contribute to different metabolic and immuno‐microenvironments during IFTA. NR1H4 can regulate the maladaptive repair of PTECs through PAH (Figure 4L). These findings provide information for further therapeutic studies on CKD.

## FUNDING INFORMATION

This study was supported by the National Natural Science Foundation of China (81970574, 82170685), as well as a grant (ZXYXZ‐201904) from the Science and Technology Commission of Shanghai Municipality, China, and a grant from Shanghai Municipal Health Commission Three‐Year Action Plan for Traditional Chinese Medicine (2021‐2023). The study was also supported by the Open Project Program Foundation of Key Laboratory of Liver and Kidney Diseases (Shanghai University of Traditional Chinese Medicine), Ministry of Education (GS2022‐01).

## CONFLICTS OF INTEREST

The authors declare no competing interests.

## Supporting information



Supporting Information

Supporting Information

Supporting Information

Supporting Information

Supporting Information

Supporting Information

Supporting Information

Supporting Information

Supporting Information

Supporting Information

Supporting Information

Supporting Information

Supporting Information

Supporting Information
