# Supplementary material for: Single‐cell RNA landscape of cell fate decision of renal proximal tubular epithelial cells and immune‐microenvironment in kidney fibrosis
Source: Clin Transl Med. 2022 Sep 9;12(9):e1010. doi: 10.1002/ctm2.1010 (PMC9460484; doi:10.1002/ctm2.1010)
Supplement: Supplementary file 14 — Supporting Information [file CTM2-12-e1010-s014.pdf]

Supplementary Material

Materials and Methods

Participants

Four patients and one transplant donor hospitalized from July 2018 to August 2018 in our hospital were prospectively enrolled. Their informed consent was acquired during enrollment after the Ethics Committee of our hospital approved the study. The study design is described in a previous study[1]. Briefly, all four patients were diagnosed with chronic kidney disease. Their fresh kidney biopsy specimens were collected for scRNA-sequencing. A flowchart of the study and the clinical characteristics of the patients are provided in Supplementary Table S1 and Supplementary Figure S1–2.

Sample preparation, cell isolation, and library preparation for scRNA-seq

Fresh kidney biopsy specimens were washed with cold PBS, put in a sterile specimen bag in an ice box, and sent to the laboratory at the earliest. The specimens were cut and enzymatically digested using a kit from Miltenyi Biotec at 37 °C. They were further strained through a filter (70 μm) with FACS buffer and centrifuged at 1,500 rpm for 10 min. The cells were resuspended with FACS buffer and strained through a filter (40 μm) to remove cell clumps. The ACK lysis buffer (Thermo) was used for erythrocyte lysis. Then, the cell suspension was washed twice using FACS buffer and assayed for cell viability after trypan blue staining with an automated cell counter (cell viability around 85%). The suspension was then loaded into the microfluidic chip on a 10X Genomics Chromium. The Illumina HiSeq platform was used for high-throughput sequencing and the sequencing library was constructed using Chromium Single Cell 3' Reagent Kits.

Quality control and preprocessing

Cell Ranger (version 2.1.1) was used for raw sequencing data processing according to the pipeline of 10X Genomics. The matrix of cell x gene was obtained based on barcode filtration according to UMI distribution. The subsequent analysis was done by Seurat (v4.0.3; http://satijalab.org/seurat/) in the R software (v4.0.2)[2]. To ensure that the results were accurate and reliable, we filtered the cells by minimum genes <300, maximum genes >7,000, hemoglobin UMI proportion > 3%, and mitochondria UMI proportion >25%[3]. The parameter of LogNormalize was set for the NormalizeData() function, along with the ScaleData () function. Finally, 21,466 cells were acquired to perform the biological analysis after the process (Supplementary Figure S3).

Integration, dimensionality reduction, and cell cluster annotation

To integrate the gene expression data of all samples, the functions of FindIntegrationAnchors and IntegrateData, developed by the Satija Lab[4], were applied. Highly variable genes (HVGs) were identified using the FindVariableFeatures() function with nfeatures = 3,000. The cell cycle effect was scored and eliminated by the ScaleData() function. Dimensionality reduction was conducted by the embedding methods of t-SNE and UMAP. The FindAllMarkers() function was used to identify differentially expressed genes (DEGs) for cell clusters compared to the others (adjusted $p < 0.05$) (Supplementary Table S2). Cell annotation was performed by referring to the DEGs, published studies, Humpheys lab website (http://humphreyslab.com/SingleCell/), and the PanglaoDB website (*https://panglaodb.se/index.html).5–9*. Then, 28 clusters were identified by Seurat, and dimensionality reduction was visualized by t-SNE and UMAP. Particularly, they were: (1) proximal tubular epithelial cells (PT), cluster 0, 1, 2, 3, 5, 6, 11, 17, and 19; (2) Endothelial cells (EC), cluster 7; (3) (Distal) connecting tubular (DCT&CNT), cluster 8; (4) Loop of Henle (LOH), cluster 9 and 21; (5) type A and B of intercalated cells, cluster 14; (6) Connecting ducts and Principal cells (CD&PC) , cluster 15; (7) Podocytes, cluster 26; (8) Pericytes, cluster 18; (9) Fibroblasts, Myofibroblasts, Pericytes, Mesangial cells (Fibro&Myofib&Peri&Mes), cluster 22; (10) Fibroblasts, cluster 23; (11) T cells, cluster 4 and 10; (12) Monocytes, cluster 12; (13) B cells, cluster 13 and 25; (14) Macrophages, cluster 16 and 20; (15) Mast cells, cluster 24; (16) Dendritic cells (DC), cluster 27 after cell-type annotation (Figure 1a-b).

Differentially expressed genes (DEGs) and enrichment analysis of function

The DEGs in the cell populations between the IFTA (IgAN, CIN) and none to mild IFTA (donor, MN, LN) groups were obtained by the FindMarkers() function with $p < 0.05$ and logfc.threshold = 0.25 (Supplementary Table 3). The DEGs of PTs were divided into “up”, “stable”, and “down” according to avg_log2FC >0, =0, or <0. Functional enrichment analysis was performed based on the GO and KEGG database by the compareCluster() function of the ClusterProfiler R package (v3.18.1)[10–12]. GSVA was also performed with all genes of PTs[13], based on the HALLMARK and KEGG databases. The AUCell package (v1.4.1) of R was used to evaluate the activity of biological pathways in the concerned cell types. For quantifying the metabolic activity in a specific cell cluster, ssGSEA[14] and scMetablism[15] were applied. The ssGSEA was performed based on all DEGs of PTs (logfc.threshold = 0.01).

Interaction analysis between cell types

To determine and visualize cell crosstalks between innate kidney cells and the immune microenvironment, the R package of CellChat[16] was used. Briefly, we loaded the Seurat object group by cell types into CellChat. Secreted signaling in CellChatDB.human was set as the reference database. The standard preprocessing steps were then applied by the standard parameter set for the identifyOverExpressedGenes, identifyOverExpressedInteractions, projectData, computeCommunProb, computeCommunProbPathway, and aggregateNet functions. Non-Negative Matrix Factorization (NMF) was used to acknowledge the communication patterns of cells.

PHATE, RNA velocity, and pseudotime trajectory analysis

PHATE (v1.0.7) was applied for nonlinear and unsupervised dimensionality reduction and visualization of cell development branches during IFTA development[17, 18]. Cells with less than 1,200 UMI/cell or over 25,000 UMI/cell, and the genes detected in fewer than 100 cells were filtered before normalization. The default parameters were used for the official pipeline. RNA velocyto was used for calculating the ratio of unspliced to spliced mRNA[19]. Transcriptional kinetics and developmental trajectory of PTs during the progression of IFTA were modeled by scVelo (v0.2.4)[20] and PAGA[21, 22] using default parameters and the filtered PT cluster from Seurat (as mentioned in *Quality control and preprocessing*). The R package of Monocle2 (v2.8.0)[23] was used for analyzing the pseudotime trajectories of PTs. Negbinomial.size was applied as the parameter expressionFamily in the newCellDataSet function to process the sparseMatrix. Low-quality cells (mean expression of genes less than 0.1) were filtered before the trajectory analysis. The SetOderingFilter() function was then used for selecting cell clustering genes with parameters of mean_expression set as 0.1 and dispersion_empirical set as 1. Dimension reduction was then performed using the function of reduceDimension with parameters of "DDRTree" and "maxcomponents" set as 2. The plotcelltrajectory() function was used for visualization after ordering cells. The analysis of branch expression analysis modeling, BEAM in Monocles package, was performed for pseudotime-related genes. The results were visualized using the plot_genes_branched_heatmap function with genes grouped by the threshold of q < 1e-4 from the BEAM analysis.

Transcription factor analysis

The R packages of SCENIC (v1.1.2.2)[24, 25], AUCell (v1.4.1), and RcisTarget(v1.2.1) were used based on the motif database for GENIE3, and RcisTarget was performed for inference of TFs-gene regulatory network. Briefly, co-expression modules were built by GENIE3 between transcription factors (TFs), genes, and the corresponding weight in the interested cell group. The RcisTarget package was used to find overrepresented TF-binding motifs for the credibility of gene regulatory networks (each TF and its target genes). Cell-type-specific transcription factors were validated using the RSS algorithm[26].

Pathology staining

Renal specimens obtained from kidney biopsy were formalin-fixed for 24 h, embedded in paraffin, and sliced into thin sections. Pathologic staining was performed after deparaffinization, rehydration, and washing with hematoxylin and eosin (HE), PAS and PASM, and Masson trichrome. Semi-quantification of deposited collagen in the Masson staining of mice kidneys was performed using Image Pro Plus (v6.0). Tubular injury scores of mice kidneys were averaged based on the percentage of injured tubules (dilatated with hypertrophy and loss of brush in the tubular epithelial cells) in 10 randomly selected cortex fields of each slide: 0, <10%; 1, 10%-25%; 2, 25%-50%; 3, 50%-75%; 4, >75% [27].

Animals

We used male Nr1h4-/-mice (8–10 weeks old) as described in our previous study[75], and sex-matched C57BL/6 wild-type mice from Jackson Lab.

Cell culture

The human PT cell line, HK-2 (ATCC CRL-2190), was cultured using Gibco™ DMEM containing bovine serum. For in vitro studies, the cells were stimulated by H_2O_2 (10 mM) for 2 h.

Folic acid model

Wild-type or Nr1h4-/-mice were injected with 0.3 M $NaHCO_3$ in the control group or 0.3 M $NaHCO_3$ + 250 mg/kg folic acid (FA) in the experiment group. Mouse kidneys were harvested seven days after injection.

Administration of the Nr1h4 inhibitor

The mice used in the experiment were orally-gavaged with Gly-β-MCA (10 mg/kg) (MCE #HY-114392) dissolved in corn oil after FA modeling every other day for one week[28], or intraperitoneally injected with Z-Guggulsterone (25 mg/kg) dissolved in corn oil (MCE #HY-110066) every day for one week[29]. The mice in

the control group were intraperitoneally provided with only corn oil after FA modelling every day for one week.

Western blotting

The tissue was homogenized in RIPA and centrifuged. The supernatant was collected and quantified using the BCA method. Then, the SDS loading buffer was added, sonication was performed, and the supernatant was boiled for 5 min, following which electrophoresis was performed using 10% SDS-PAGE gels. The samples were then transferred onto a PVDF membrane and blocked by BSA. The samples were incubated with the primary antibodies (anti-rabbit PAH, Abcam, #ab191415; anti-mouse NR1H4/FXR, CST, #72105S; anti-Rabbit HNF4A, Abcam, #ab200142; anti-mouse BAX, Proteintech, #60267–1-Ig; anti-rabbit CASPASE-3, CST, Cat#9662; anti-mouse BCL2, CST,#15071S) and the corresponding secondary antibodies. Membrane development was performed using the HRP Substrate Kit from Millipore and imaged using ImageReader LAS-4000 (Fujifilm).

QPCR

We isolated the total RNA from the kidney cortex with TRIzol and quantitated it by performing UV spectrophotometry. The RNA (1 ng) was then used to synthesize cDNA using a kit from Tiangen (#KR118). The expression of the mRNA was assessed by performing qPCR using TB Green® Premix Ex Taq™ (TaKaRa, #RR420A).

Small interfering RNA (siRNA) transfection

The siRNAs targeting PAH were provided by Genomeditech and added to HK-2 cells at a final concentration of 100 nM 24 h before they were treated with H_2O_2 using Lipofectamine3000 from Thermo (#L3000150). The sequences (5'-3') of the siRNAs used were as follows: PAH(h)-si-2, CCUGACAUCUGCCAUGAGC tt, GCUCAUGGCAGAUGUCAGG tt; PAH(h)-si-3, UCGCAUUUCAUCAAGAUUAAU tt, and AUUAAUCUUGAUGAAAUGCGA tt.

Flow cytometry

The HK2 cells with or without H_2O_2 modeling and stained with Annexin-PI were analyzed through flow cytometry (BD Pharmingen™, #556547).

Statistical analysis

SPSS v26.0 (IBM), Prism v9.0 (GraphPad), and R v4.0.5 were used for conducting the statistical analysis of the clinical cohort data. Differential analysis between the two clinical groups was conducted by performing the Student's t-test or Mann-Whitney U test. The differences were considered to be statistically significant at $p < 0.05$.

External data validation

The renal scRNA-seq data on IgA nephropathy with IFTA grade (GSE127136)[30]and snRNA-seq data on diabetic nephropathy with IFTA grade (GSE131882)[31] were downloaded from the GEO database. Integration, dimensionality reduction, cell cluster annotation, and downstream analysis by Seurat were performed following the same method as mentioned above.

References

1. Chen Z, Zhang T, Mao K, Shao X, Xu Y, Zhu M, et al. A single-cell survey of the human glomerulonephritis. J Cell Mol Med. 2021;25(10):4684-95.
2. Butler A, Hoffman P, Smibert P, Papalexi E, Satija R. Integrating single-cell transcriptomic data across different conditions, technologies, and species. Nat Biotechnol. 2018;36(5):411-20.
3. Balzer MS, Ma Z, Zhou J, Abedini A, Susztak K. How to Get Started with Single Cell RNA Sequencing Data Analysis. J Am Soc Nephrol. 2021;32(6):1279-92.
4. Stuart T, Butler A, Hoffman P, Hafemeister C, Papalexi E, Mauck WM, 3rd, et al. Comprehensive Integration of Single-Cell Data. Cell. 2019;177(7):1888-902 e21.
5. Park J, Shrestha R, Qiu C, Kondo A, Huang S, Werth M, et al. Single-cell transcriptomics of the mouse kidney reveals potential cellular targets of kidney disease. Science. 2018;360(6390):758-63.
6. Stewart BJ, Ferdinand JR, Young MD, Mitchell TJ, Loudon KW, Riding AM, et al. Spatiotemporal immune zonation of the human kidney. Science. 2019;365(6460):1461-6.
7. Muto Y, Wilson PC, Ledru N, Wu H, Dimke H, Waikar SS, et al. Single cell transcriptional and chromatin accessibility profiling redefine cellular heterogeneity in the adult human kidney. Nat Commun. 2021;12(1):2190.
8. Kuppe C, Ibrahim MM, Kranz J, Zhang X, Ziegler S, Perales-Paton J, et al. Decoding myofibroblast origins in human kidney fibrosis. Nature. 2021;589(7841):281-6.
9. Kirita Y, Wu H, Uchimura K, Wilson PC, Humphreys BD. Cell profiling of mouse acute kidney injury reveals conserved cellular responses to injury. Proc Natl Acad Sci U S A. 2020;117(27):15874-83.
10. Yu G, Wang LG, Han Y, He QY. clusterProfiler: an R package for comparing biological themes among gene clusters. OMICS. 2012;16(5):284-7.
11. Kanehisa M, Goto S. KEGG: kyoto encyclopedia of genes and genomes. Nucleic Acids Res. 2000;28(1):27-30.
12. Harris MA, Clark J, Ireland A, Lomax J, Ashburner M, Foulger R, et al. The Gene Ontology (GO) database and informatics resource. Nucleic Acids Res. 2004;32(Database issue):D258-61.
13. Hanzelmann S, Castelo R, Guinney J. GSVA: gene set variation analysis for microarray and RNA-seq data. BMC Bioinformatics. 2013;14:7.

14. Foroutan M, Bhuva DD, Lyu R, Horan K, Cursons J, Davis MJ. Single sample scoring of molecular phenotypes. BMC Bioinformatics. 2018;19(1):404.
15. Wu Y, Yang S, Ma J, Chen Z, Song G, Rao D, et al. Spatiotemporal Immune Landscape of Colorectal Cancer Liver Metastasis at Single-Cell Level. Cancer Discov. 2022;12(1):134-53.
16. Jin S, Guerrero-Juarez CF, Zhang L, Chang I, Ramos R, Kuan CH, et al. Inference and analysis of cell-cell communication using CellChat. Nat Commun. 2021;12(1):1088.
17. Moon KR, van Dijk D, Wang Z, Gigante S, Burkhardt DB, Chen WS, et al. Visualizing structure and transitions in high-dimensional biological data. Nat Biotechnol. 2019;37(12):1482-92.
18. Fan Y, Min Z, Alsolami S, Ma Z, Zhang E, Chen W, et al. Generation of human blastocyst-like structures from pluripotent stem cells. Cell Discov. 2021;7(1):81.
19. La Manno G, Soldatov R, Zeisel A, Braun E, Hochgerner H, Petukhov V, et al. RNA velocity of single cells. Nature. 2018;560(7719):494-8.
20. Bergen V, Lange M, Peidli S, Wolf FA, Theis FJ. Generalizing RNA velocity to transient cell states through dynamical modeling. Nat Biotechnol. 2020;38(12):1408-14.
21. Wolf FA, Hamey FK, Plass M, Solana J, Dahlin JS, Gottgens B, et al. PAGA: graph abstraction reconciles clustering with trajectory inference through a topology preserving map of single cells. Genome Biol. 2019;20(1):59.
22. Hota SK, Rao KS, Blair AP, Khalilimeybodi A, Hu KM, Thomas R, et al. Brahma safeguards canalization of cardiac mesoderm differentiation. Nature. 2022;602(7895):129-34.
23. Qiu X, Mao Q, Tang Y, Wang L, Chawla R, Pliner HA, et al. Reversed graph embedding resolves complex single-cell trajectories. Nat Methods. 2017;14(10):979-82.
24. Marx V. May mechanobiology work forcefully for you. Nat Methods. 2019;16(11):1083-6.
25. Davie K, Janssens J, Koldere D, De Waegeneer M, Pech U, Kreft L, et al. A Single-Cell Transcriptome Atlas of the Aging Drosophila Brain. Cell. 2018;174(4):982-98 e20.
26. Suo S, Zhu Q, Saadatpour A, Fei L, Guo G, Yuan GC. Revealing the Critical Regulators of Cell Identity in the Mouse Cell Atlas. Cell Rep. 2018;25(6):1436-45 e3.
27. Okubo K, Kurosawa M, Kamiya M, Urano Y, Suzuki A, Yamamoto K, et al. Macrophage extracellular trap formation promoted by platelet activation is a key mediator of rhabdomyolysis-induced acute kidney injury. Nat Med. 2018;24(2):232-8.
28. Jiang C, Xie C, Lv Y, Li J, Krausz KW, Shi J, et al. Intestine-selective farnesoid X receptor inhibition improves obesity-related metabolic dysfunction. Nat Commun. 2015;6:10166.
29. Liu M, Wang W, Wang J, Fang C, Liu T. Z-Guggulsterone alleviates renal fibrosis by mitigating G2/M cycle arrest through Klotho/p53 signaling. Chem Biol Interact. 2022;354:109846.
30. Zheng Y, Lu P, Deng Y, Wen L, Wang Y, Ma X, et al. Single-Cell Transcriptomics Reveal Immune Mechanisms of the Onset and Progression of IgA Nephropathy. Cell Rep. 2020;33(12):108525.
31. Wilson PC, Wu H, Kirita Y, Uchimura K, Ledru N, Rennke HG, et al. The single-cell transcriptomic landscape of early human diabetic nephropathy. Proc Natl Acad Sci U S A. 2019;116(39):19619-25.

Supplementary Figures

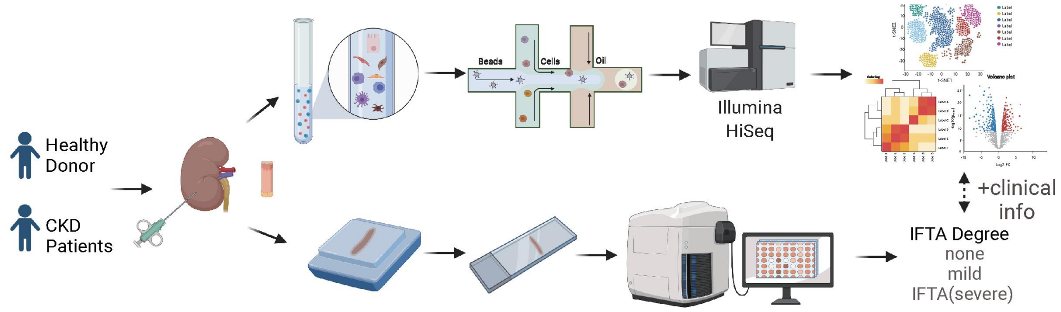

Supplementary Figure S1. Flow chat of the study.

IFTA, interstitial fibrosis and tubular atrophy; CKD, chronic kidney disease; The scheme was drawn using BioRender.com.

a

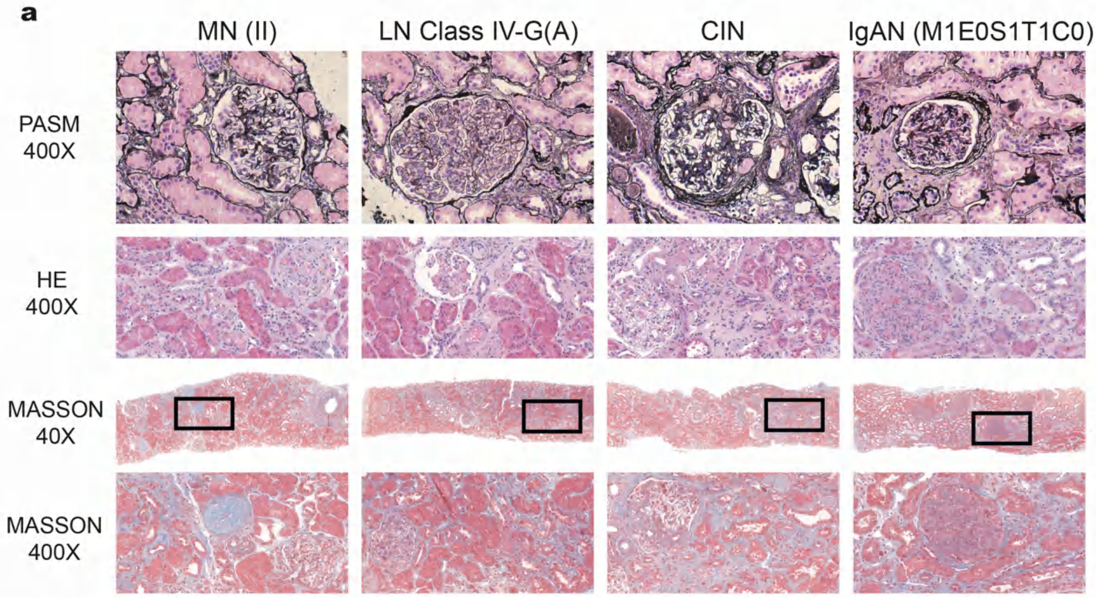

b

	Light microscopy			IF
	Glomerulus	IFTA	Vasculature	
MN	28 glomeruli. The capillary loops of each glomerulus were slightly irregularly thickened. There were vacuole-like changes and spike formation under epithelium by PASM staining. The double-track sign was occasionally observed. Mesangial cells and stromal were segmentally increasd, occasionally infiltrated with inflammatory cell	mild	thickened arteriole intima, segmental hyaline degeneration	PLA2R（+）;IgG++, IgA+, IgM±, C3+, C1q±~+, κ++, λ++
CIN	34 glomeruli, 14(41%) of which showed globular sclerosis, and some glomerular mesangial cells and stromal segments were slightly increased, and some glomerular capillary loops showed ischemic shrinkage with encapsulation and thickened capsule wall	moderate to severe	thickened and sclerotic arteriole intima, segmental hyaline degeneration	IgG-, IgA-, IgM-, C3-, C1q-, κ-, λ-
LN	11 glomeruli. Some were enlarged and lobulated. The mesangial cells and stroma of each glomeruli were obviously increased. There were diffuse capillary proliferation and inflammatory cell infiltration. There were small crescents(mainly cellular) in 3(27%)glomeruli and adhered to the capillary loops. The capillary loops were slightly thickened, and eosinophilic complex deposits can be seen under the endothelium	mild	/	IgG+++, IgA++, IgM±, C3++, C1q+~++, κ++, λ++
IgAN	13 glomeruli, 4(30%) of which showed globular sclerosis, and 2 (15%) segmental sclerosis. The mesangial cells and stromal cells in the remaining glomeruli were slightly increased	moderate to severe	segmental thickened arteriole intima, hyaline degeneration of (interlobar)arterioles	IgG-, IgA+++, IgM-, C3±, C1q-, κ++, λ+++,

Supplementary Figure S2. Pathology of participants

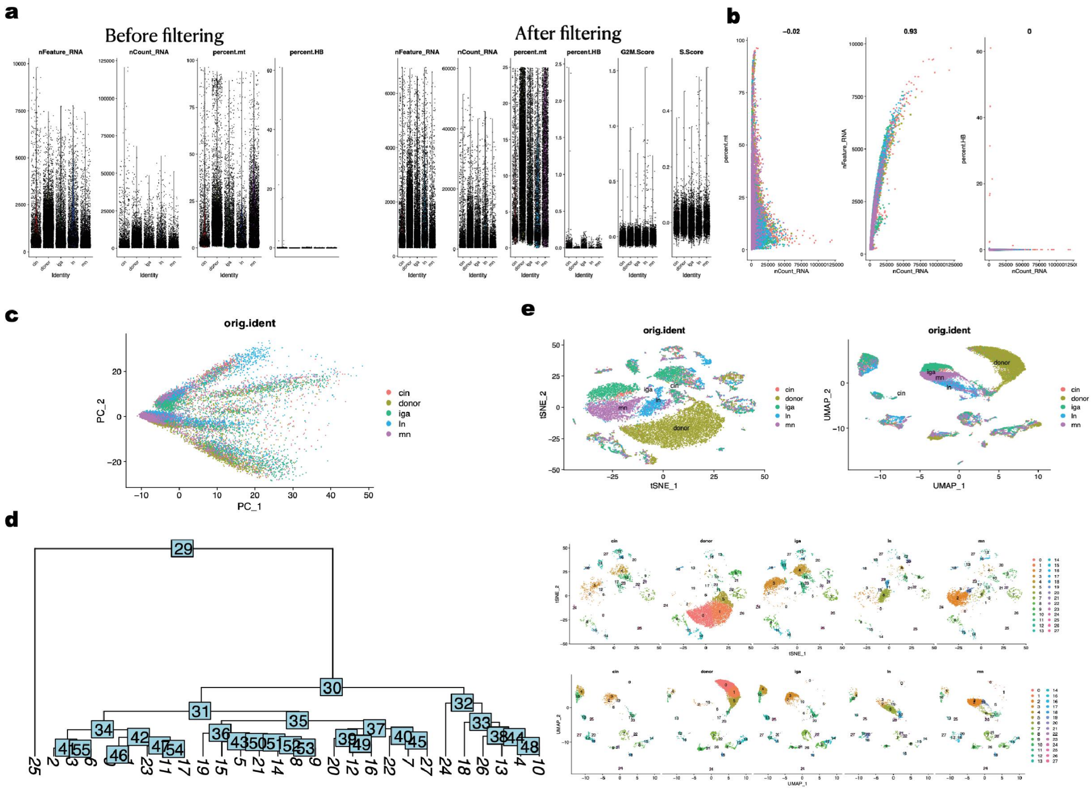

Supplementary Figure S3. Pre-process and quality control of the scRNA-seq data.

a-b, quality control of the cells and genes for analyzing;c, the integrative dimensionality reduction of cells from different samples by PCA; e, unbiased clustering of cells from all samples by t-SNE (top left) and UMAP (top right) annotated with sample names. Unbiased clustering of cells split by different samples by t-SNE annotated with Seurat clusters; d, Phylogenetic Analysis of Identity Seurat clusters.

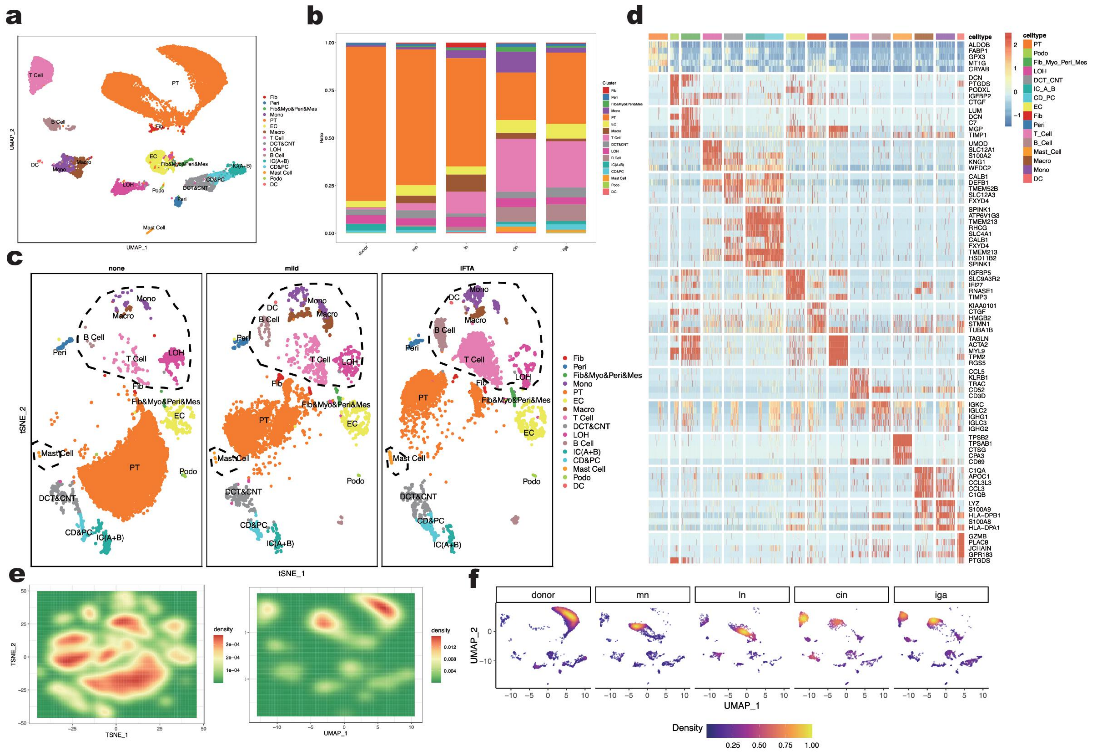

Supplementary Figure S4. Detailed cell proportion and top 5 cluster marker genes of the scRNA-seq data.

a, unbiased clustering of cells from all samples by UMAP; b, relative cell proportion across 5 kidney samples; c, unbiased clustering of cells in each IFTA grade by tSNE; d, heatmap of the expression of top10 markers of cell type; e, density plot of cells in different clusters based on tSNE and UMAP. PTs and T cells were the top 2 dense clusters among all; f, density plot of cells in different clusters based on tSNE in each sample, IFTA samples (CIN and IgAN) had densely clustered PTs (Seurat cluster 2, 3) and T cells compared to mild (MN and LN) or none (Donor) samples.

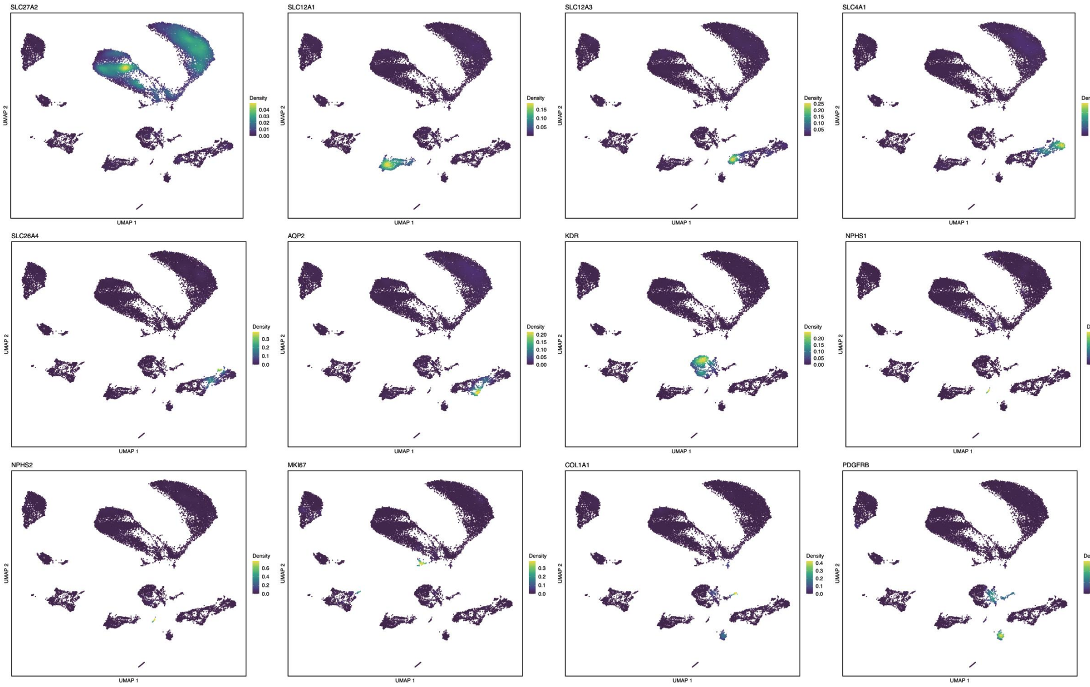

Supplementary Figure S5. Density plot of makers for non-immune cells in kidney

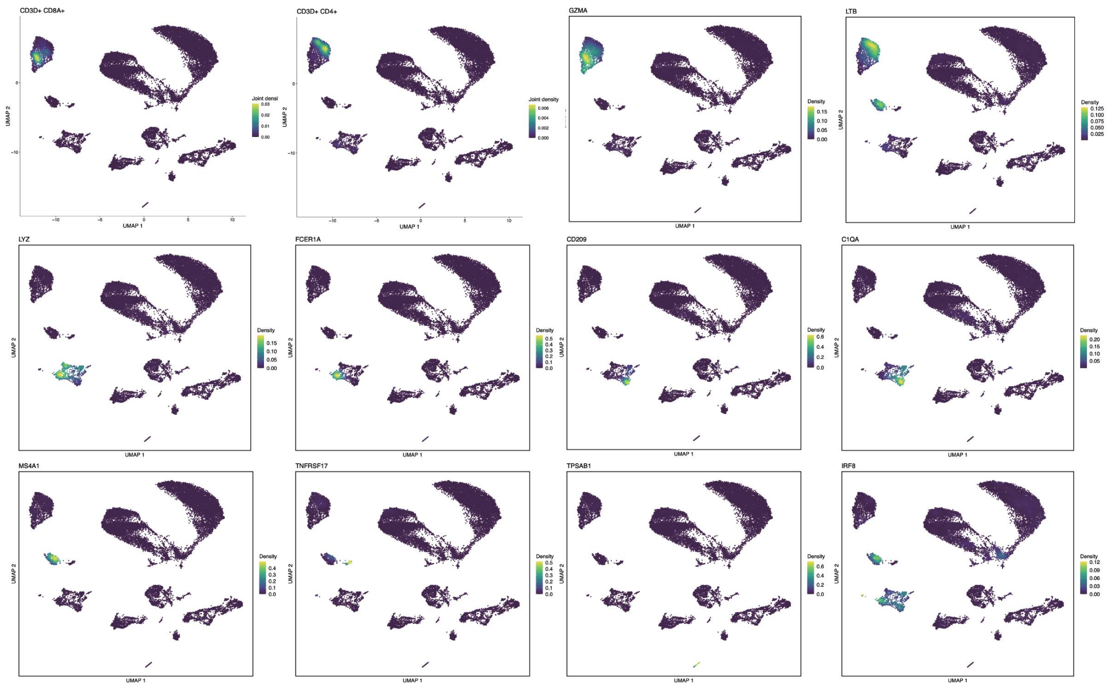

Supplementary Figure S6. Density plot of makers for immune cells in kidney

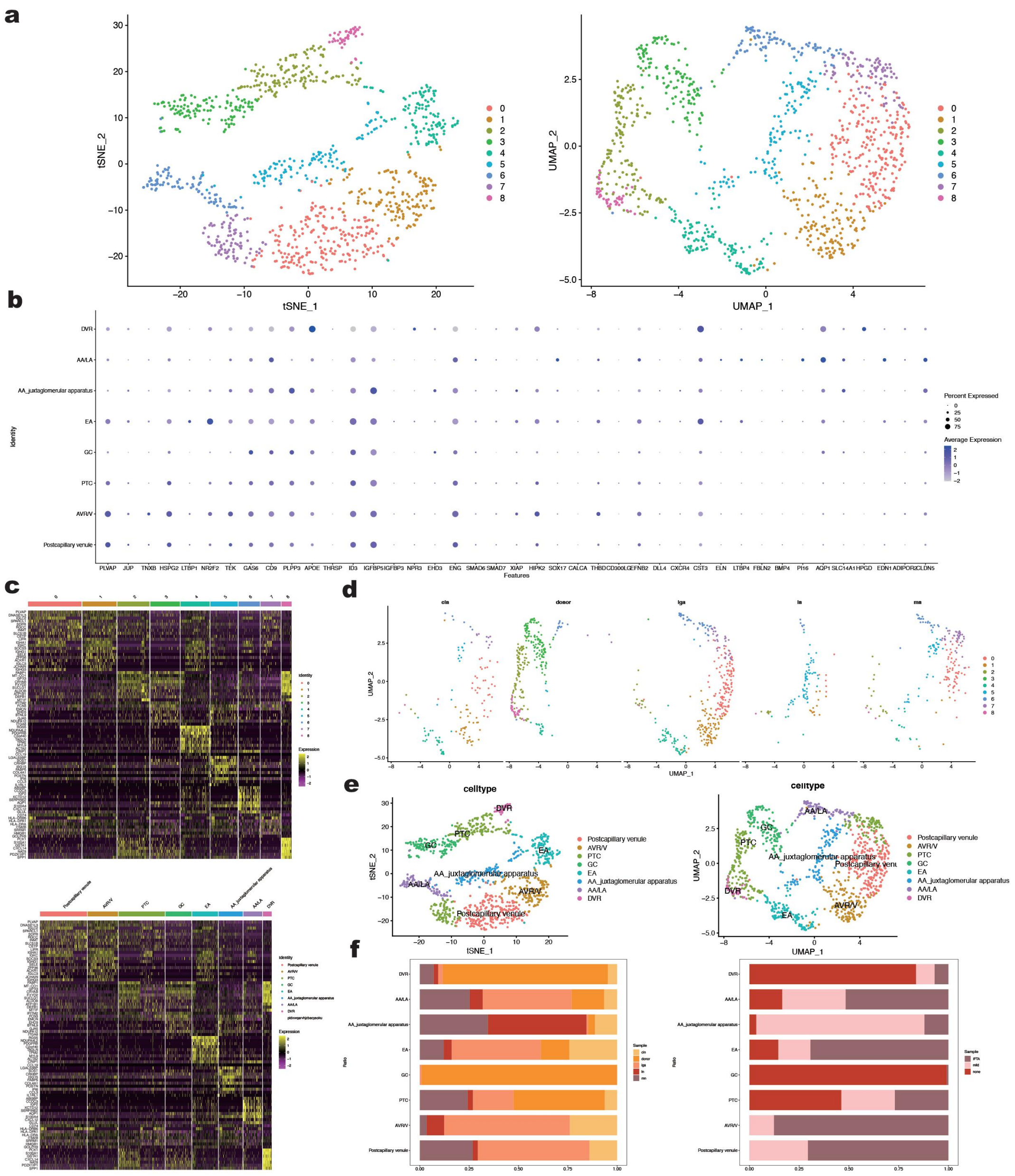

Supplementary Figure S7. Subcluster of Endothelial cells in kidney IFTA

a, clustering of endothelial cells from all samples by t-SNE and UMAP; b, dot plots of the markers' expression specific for cell populations; c, heatmap of the markers' expression specific for cell populations; d, clustering of endothelial cells across samples; e, annotated unbiased clustering of cells from all samples by t-SNE and UMAP; f, relative cell proportion across 5 kidney samples.

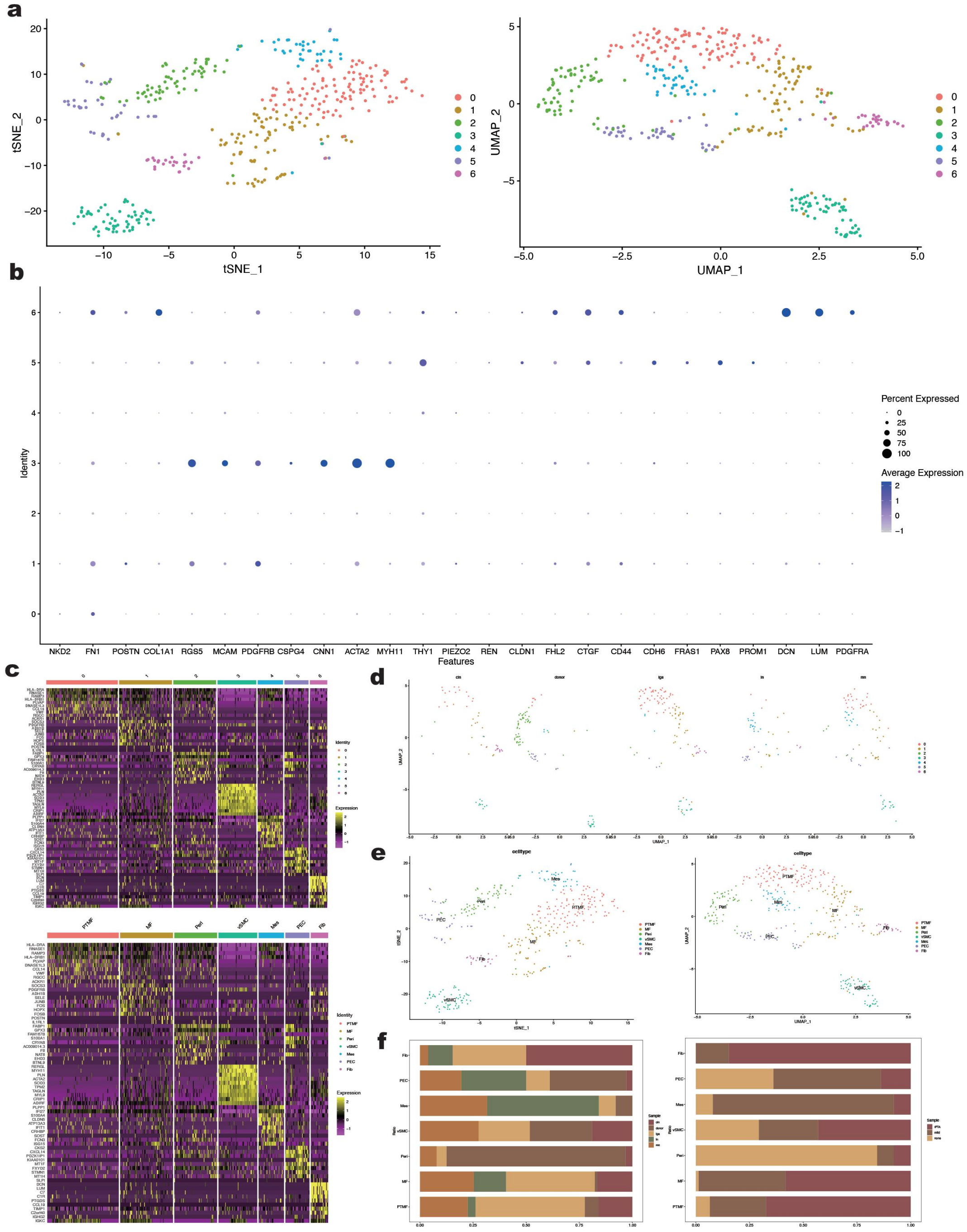

Supplementary Figure S8. Subcluster of (myo)fibroblast-like cells in kidney IFTA

a, clustering of (myo)fibroblast-like cells from all samples by t-SNE and UMAP; b, dot plots of markers' expression specific for cell populations; c, heatmap of the markers' expression specific for cell populations; d, clustering of (myo)fibroblast-like cells in each sample; e, annotated unbiased clustering of cells from all samples by t-SNE and UMAP; f, relative cell proportion across 5 kidney samples.

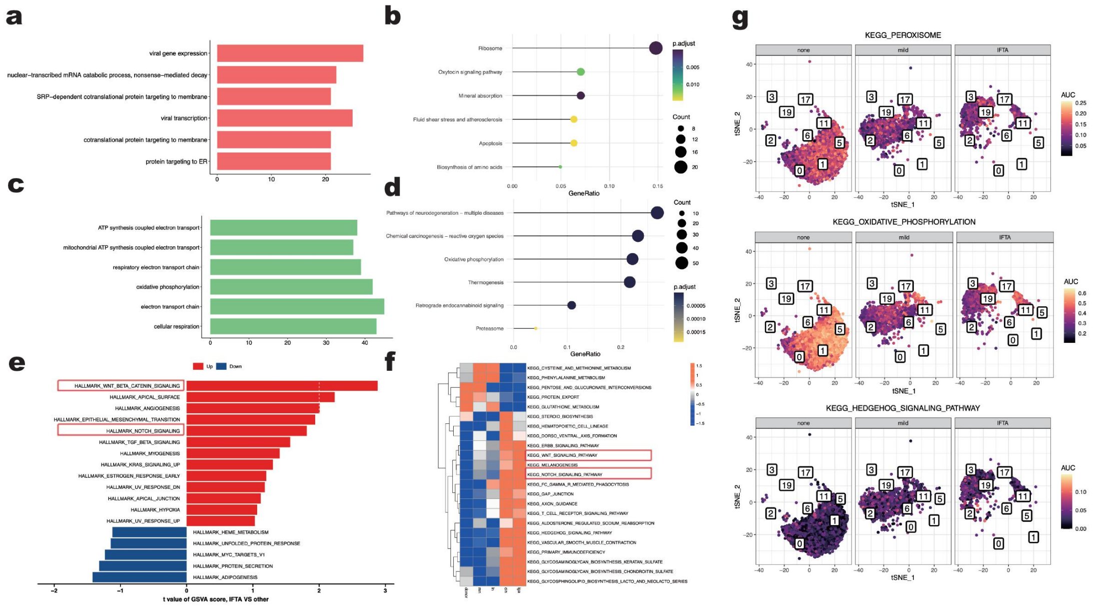

Supplementary Figure S9. Detailed enriched pathway based on DEGs among PTs in IFTA vs other group

a-b, top 6 changed pathways by enrichment analysis using up-regulated DEGs of PTs ($p<0.05$, log2FC>0.25) based on GO Biological Process database (a) and KEGG database (b); c-d, top 6 changed pathways by enrichment analysis using down-regulated DEGs of PTs ($p<0.05$, log2FC<-0.25) based on GO Biological Process database (c) and KEGG database (d); e, GSVA result of pathway activity in IFTA group compared to others visualized by bar plot. The analysis was based on HALLMARKER of MSigDB database; f, GSVA result of pathway activity across different samples visualized by heatmap. The analysis was based on KEGG of MSigDB database; g, AUCell visualizing the relatively down-regulating activity of peroxisome and oxidative phosphorylation pathways and up-regulating activity of cell proliferation-related hedgehog pathways with IFTA progression.

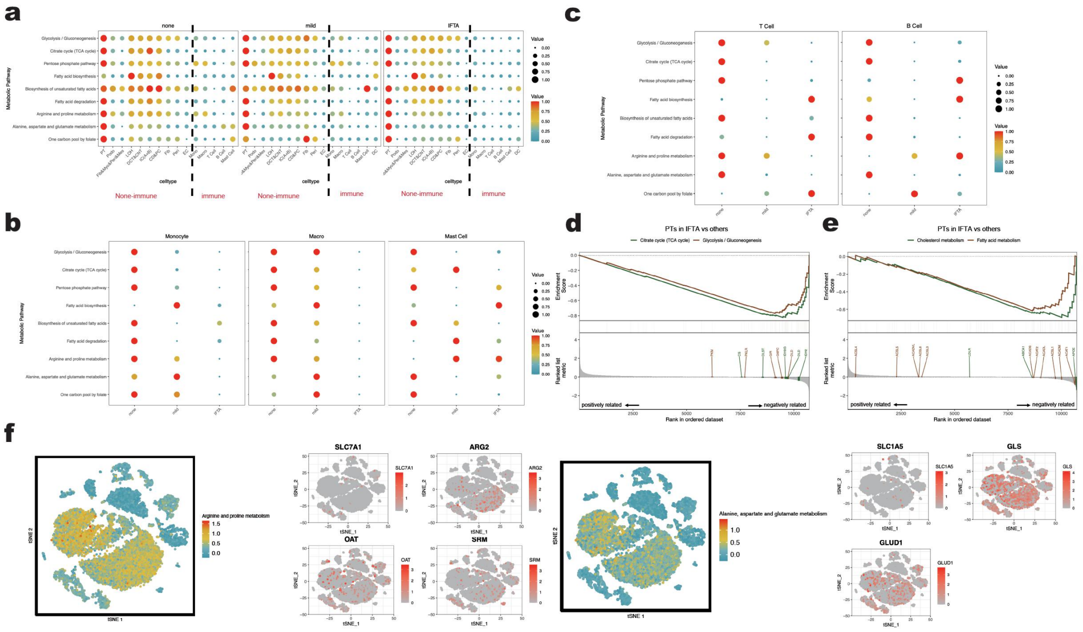

Supplementary Figure S10. Metabolic change among cell clusters in IFTA group vs others

a, The overall metabolic pathway activity score of each cell cluster in different IFTA groups by scMetabolism method. **b,** The metabolic pathway activity score of immunes cells of myeloid lineage by scMetabolism method; **c,** The metabolic pathway activity score of immunes cells of lymphoid lineage by scMetabolism method; d-e, ssGSEA analysis of DEGs in PTs among IFTA group versus others by FindMarker function with parameters min.pct setting at 0.01 and logfc.threshold setting at 0.01. The analysis was performed based on KEGG database. Detailed DEGs and enriched pathways were in Supplementary Table S4-5. (d), TCA cycle pathway (NES=-2.0602, adj.p=0.0000) and glycolysis/gluconeogenesis pathway (NES=-2.1135, adj.p=0.0000). Interested genes encoding key enzymes of TCA cycle (CS, IDH3G, IDH2, OGDH, DLST, DLD), or glycolysis (HK, GPI, G6PC, PKLR, PKM) were marked according to their correlation to the pathway if they were in the DEGs; (e), pathway of fatty acid metabolism (NES, -1.7087; adj.p, 0.0091) and cholesterol metabolism (NES, -1.7918; adj.p, 0.0065). Interested genes encoding key enzymes of fatty acid activation (ACSS1-3, ACSL1, ACSL3-6, SLC27A2, ACSF2-3, ACSM2A-2B, ACSM1, ACSM3-6), fatty acid transport (CROT, CRAT, CPT1), fatty acid β-oxidation (ACAD family, -S, -M, -L, -VL), cholesterol biosynthesis (SREBP1, SREBP2, HMGCR), cholesterol uptake (NPC1L1, LDLR), cholesterol esterification (ACAT1, ACAT2), cholesterol export (ABCA1, ABCG1, ABCG5, ABCG8, NR1H3, NR1H2, APOE) were marked according to their correlation to the pathway if they were in the DEGs; f, dimplot of arginine and glutamate pathway activity among all samples by scMetabolism method, and feature plot of genes encoding related key enzymes on the right. The dot position represented the localization of genes. The dot color represented the expression of genes.

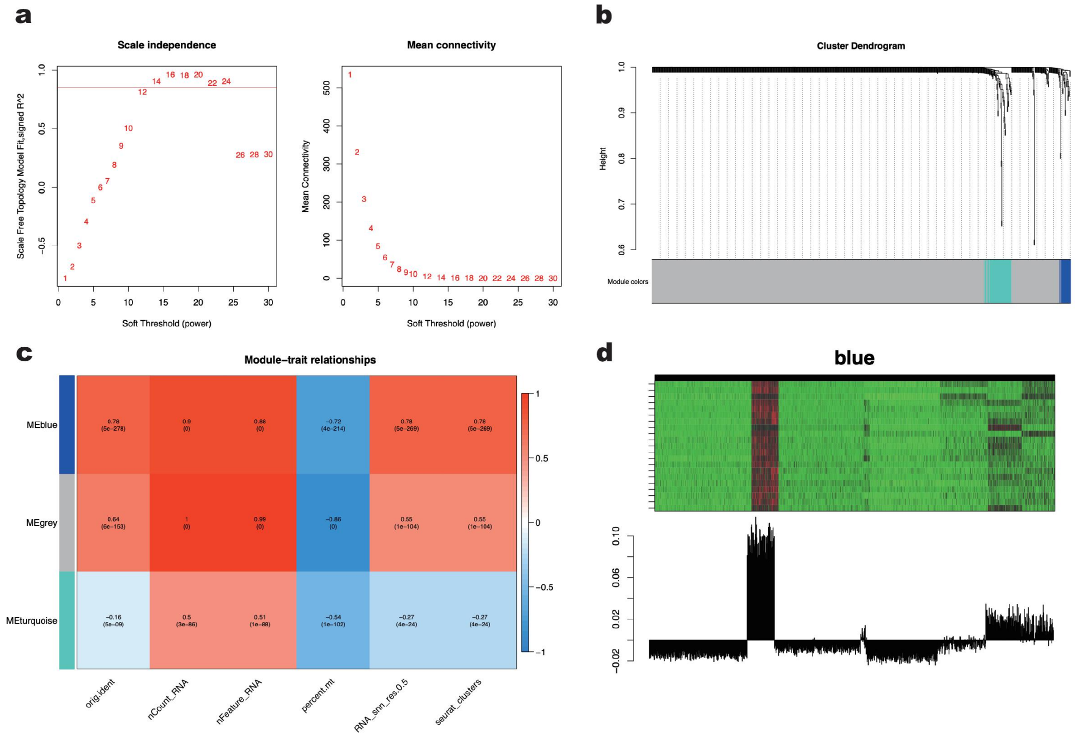

Supplementary Figure S11. Weighted Gene Correlation Network analysis and identification of the key module for transcriptional heterogeneity of renal tubular epithelial cells

a, The parameter β representing thresholding powers was set as 14 for further scale-free topology; b, Gene dendrogram of hierarchically clustered different genes estimated by topologic overlap. The color row below indicated the modules determined by the clustering tree; c, Correlation analysis between the module and the sample features. Module eigengene was represented by row while sample features by columns. The correlation coefficient and p-value were presented inside each color block. The royalblue module was significantly correlated to pathology type, IFTA (orig.ident); d, Heatmap demonstrating the distribution of genes expression in the module in royalblue.

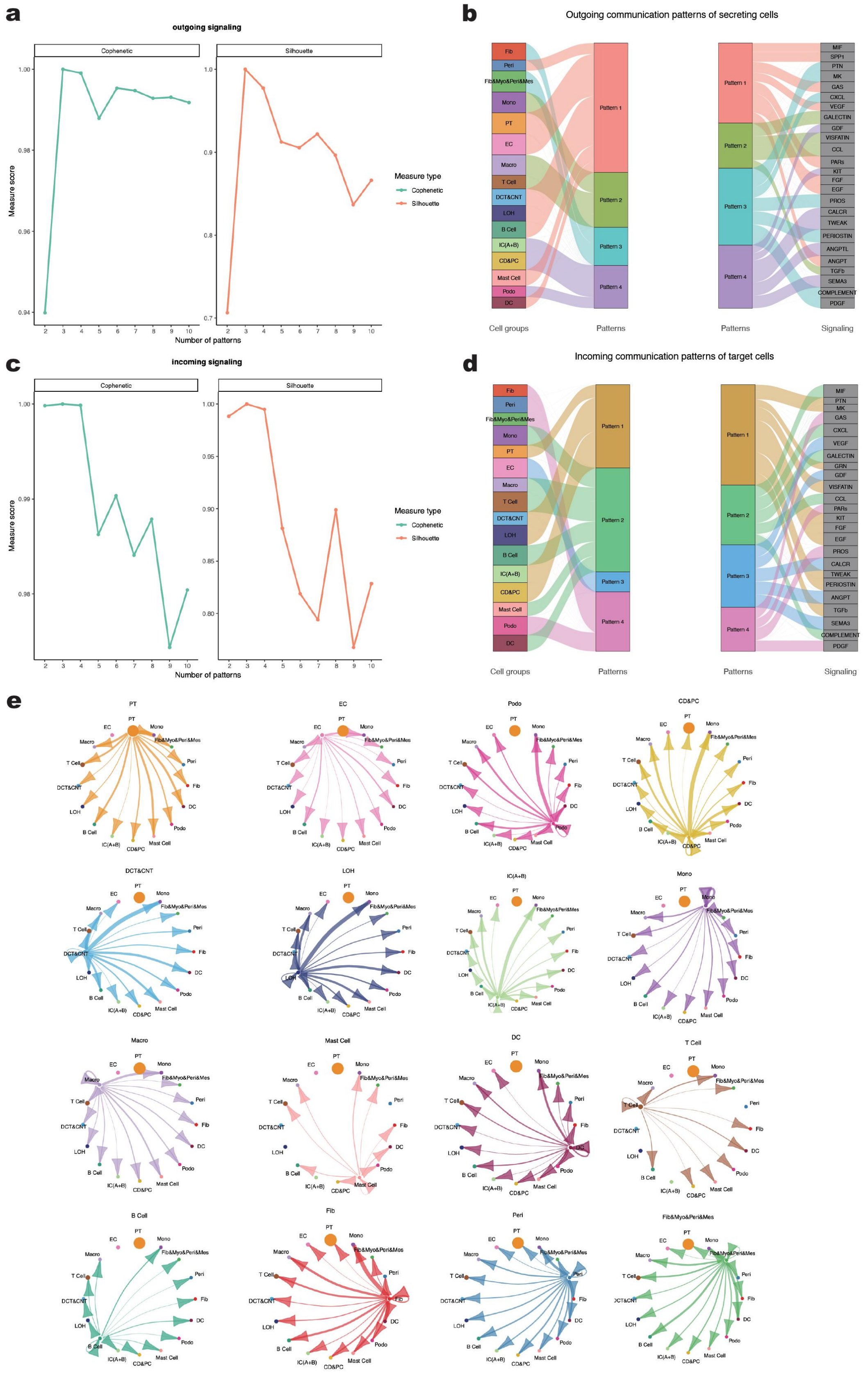

Supplementary Figure S12. Overall cell-to-cell communications among innate kidney cells and the infiltrated immune cells

a-b, the inferred patterns of the outgoing signaling pathways from cells demonstrated the communication between patterns, cell clusters, and signaling pathways analyzed by NMF method. K=4 for NMF calculation according to (a) where the first low point was chosen; The patterns could be subclustered into 4 types, and the weight of single cell population or pathway to the latent patterns was reflected by the thickness of the flow(b). c-d, the plot structures of inferred incoming communication patterns of cells. K=4 for NMF calculation according to (c) where the first low point was chosen; The patterns could be subclustered into 4 types, and PTs were in the same cluster as the other tubule cells DCT&CNT, LOH, IC(A+B), CD&PC; e, circle plot summarizing the weight/strength of the interactions from one cell type to the others among all samples. The thickness of the arrows represents communication strength and the arrow direction represents the direction of the signaling pathway. PTs secret signaling pathway to almost all cells (strongest communication to immune cells) while receiving signaling pathway mainly from (myo)fibroblast-like cells.

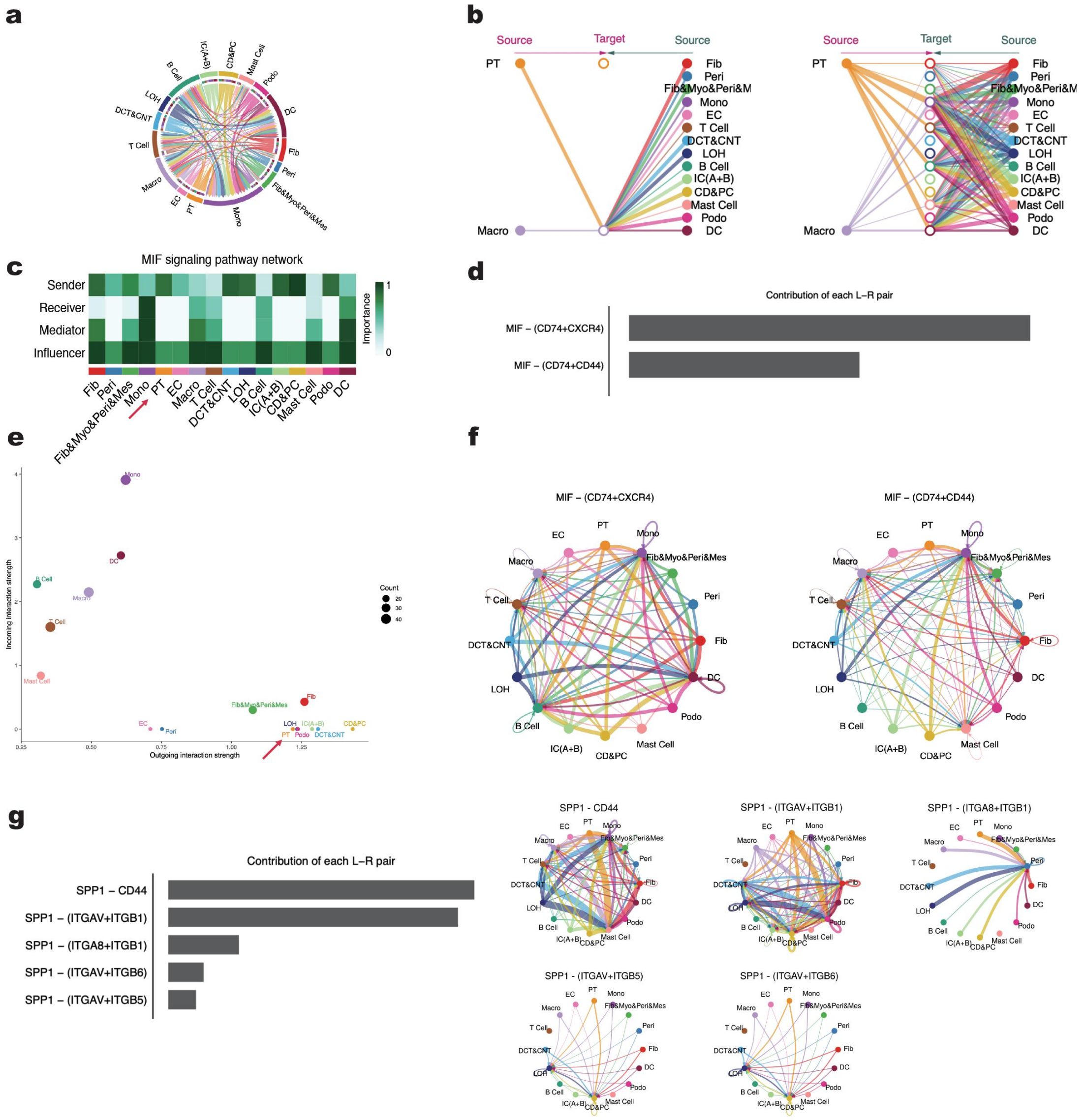

Supplementary Figure S13. Main signaling pathways between PT and the infiltrated immune cells

a, summary chord plots showing the overall communication network of MIF signaling pathway, which was one of the main pathways between PT and the infiltrated immune cells; b, hierarchical plot illustrating the crosstalk through MIF signaling pathway between PTs, macrophages, and other types of cells. The left portion highlighted PT and macrophage as signaling targets in the mild while the right proportion highlighted the other cells as signaling targets. Solid circles represent the source while open as the target. The cell number in each population was represented by the size of circles. The communication probability was represented by the edge width; c-d, clustering heatmap (c) and bubble plot (d) of each cell population's role in MIF signaling pathway; e, bar plot indicating the proportion of ligand-receptor (L-R) pairs in MIF signaling pathway; f, circle plots summarizing each L-R pair of MIF signaling among cell types; g, bar plot indicating the proportion of L-R pairs in signaling of SPP1 and circle plots summarizing L-R pairs in signaling of SPP1 among cell types.

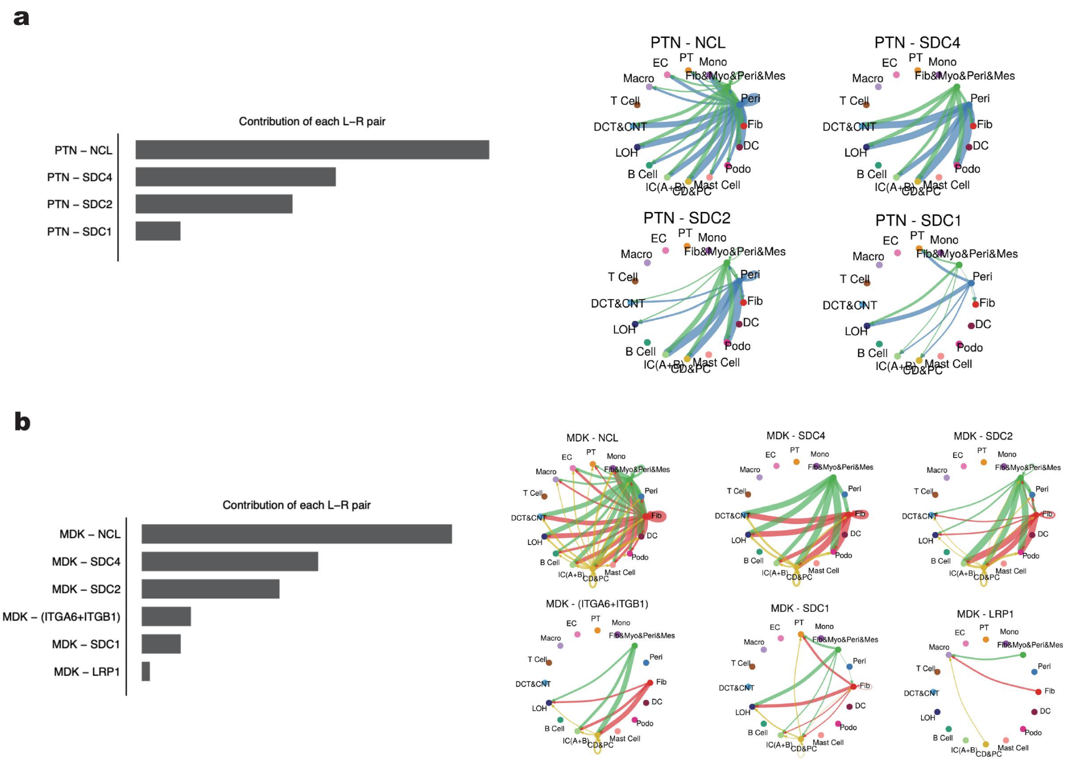

Supplementary Figure S14. Main signaling pathways to PT

a, bar plot indicating the proportion of ligand-receptor (L-R) pairs in PTN signaling pathway to PTs and circle plots summarizing each L-R pair in PTN signaling among cell types; b, bar plot indicating the proportion of ligand-receptor (L-R) pairs in MK signaling pathway to PTs and circle plots summarizing each L-R pair in MK signaling among cell types.

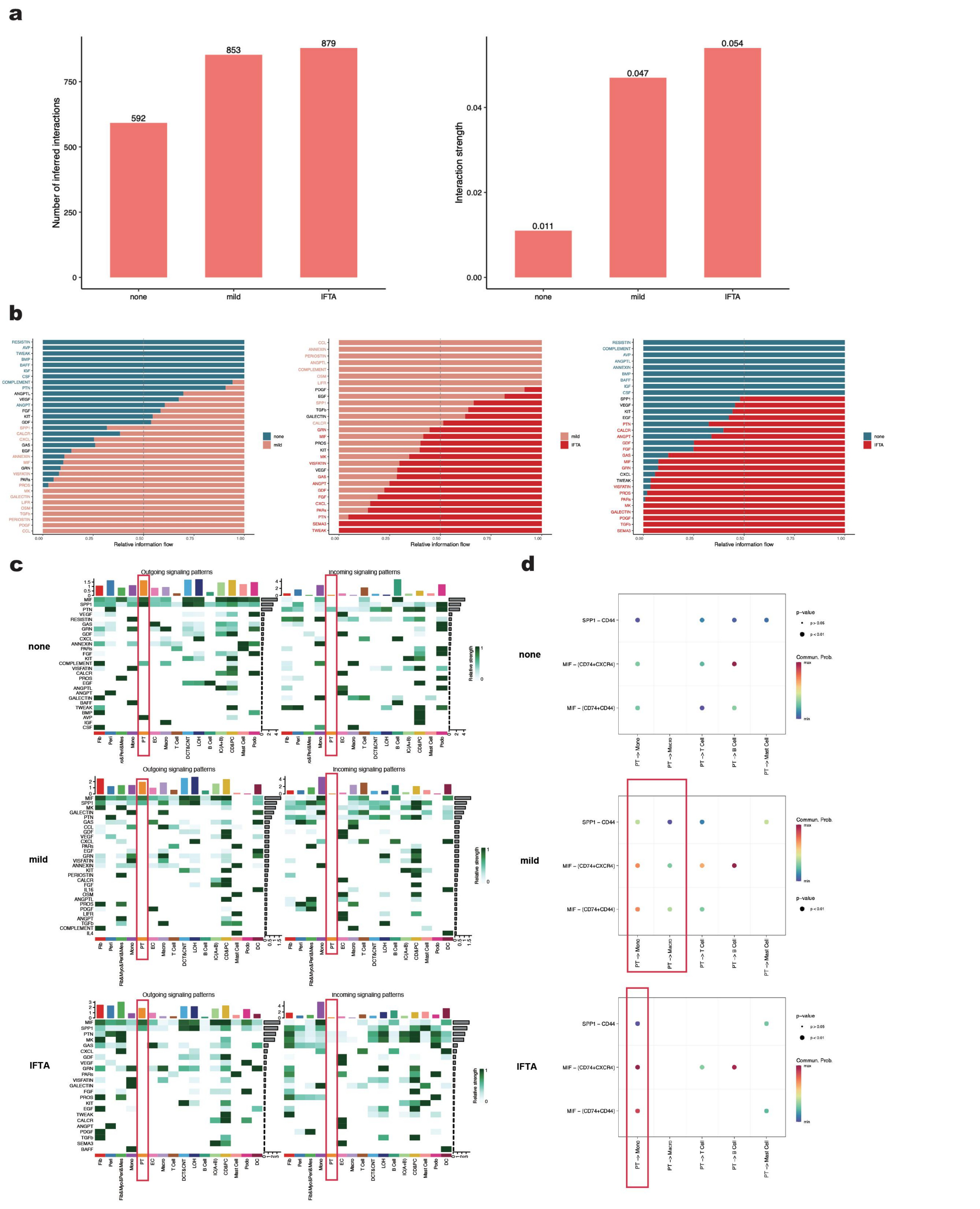

Supplementary Figure S15. Different cell-to-cell communications pathways among different IFTA kidney

a, bar plot indicating the comparison of cell interactions in samples acorss different IFTA groups in the aspect of numer(left) and weight/strength(right); b, bar plot indicating relative cell-to-cell interaction strength of each signaling pathway in none vs mild (left), mild vs IFTA (middle), none vs IFTA (right) group; c, summarizing heatmap showing the outgoing (secreting) and incoming (target) signal patterns in different

IFTA groups; d, bubble plots of the main signaling pathway from PT to infiltrated immune cells in different IFTA groups.

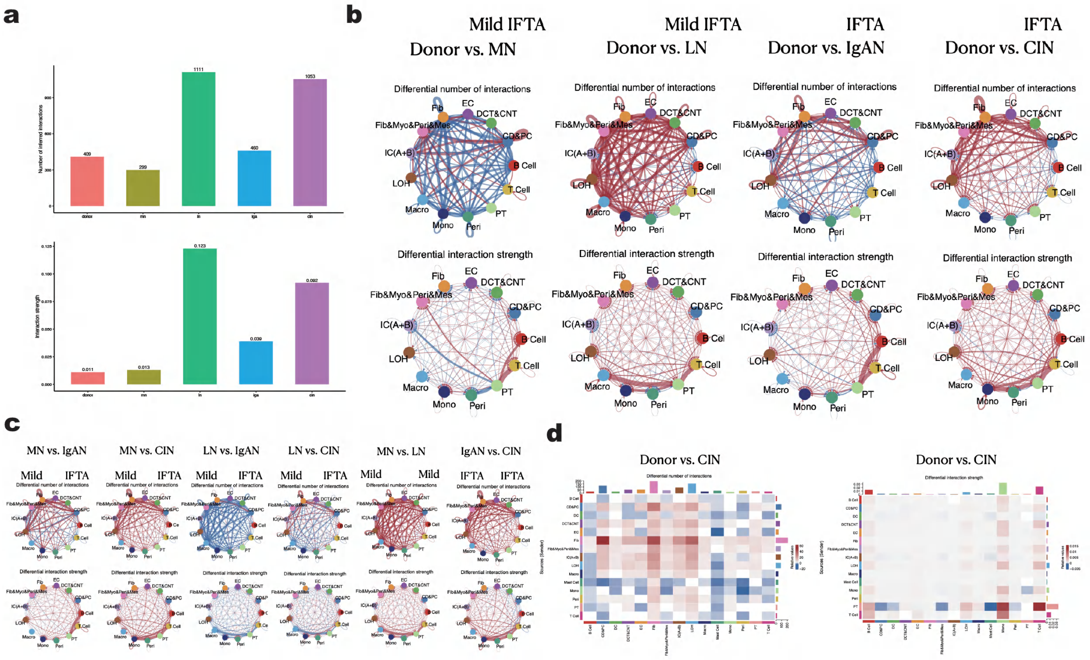

Supplementary Figure S16. Detailed Comparison of cell-to-cell communications among various samples

a, bar plot indicating the comparison of cell interactions in samples acorss different samples in the aspect of number(left) and weight/strength(right); b-c, circle plots summarizing the number (up) and weight/strength of the crosstalks (down) across different samples with different IFTA degrees; d, summarizing heatmap indicating number (left) and weight/strength of the interactions (right) between the donor and cin kidney.

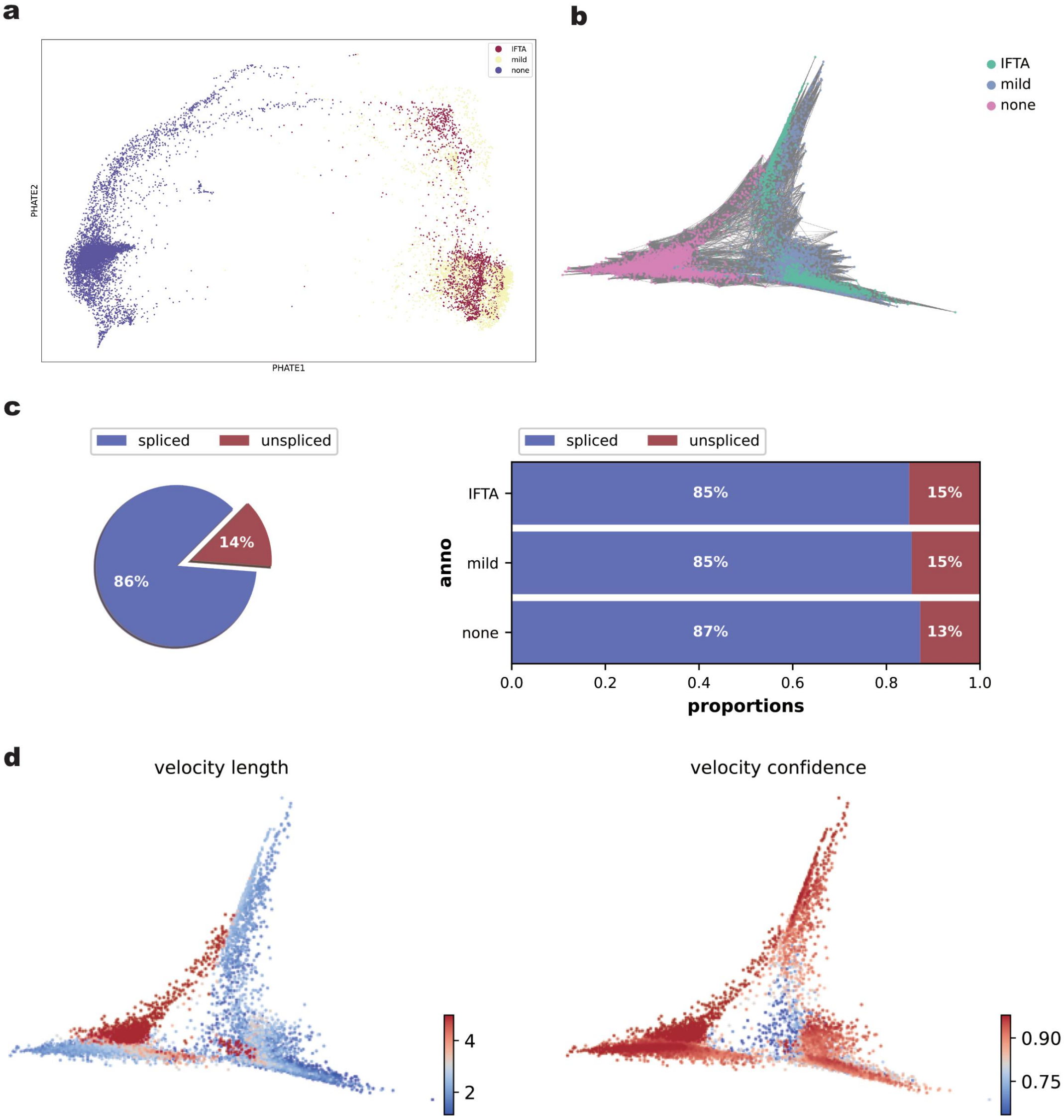

anno	IFTA	mild	none
velocity_length	1.720836	1.880015	2.532766
velocity_confidence	0.746314	0.824582	0.926233

seurat_clusters	0	1	2	3	5	6	11	17	19
velocity_length	2.115641	1.982888	1.664061	1.585490	4.577583	2.060456	3.168925	1.830659	2.735177
velocity_confidence	0.905013	0.937313	0.800741	0.715268	0.954348	0.848998	0.892749	0.906845	0.706376

Supplementary Figure S17. PHATE and RNA velocity recapitulates dynamics of PT cell differentiation

a, 2D PHATE embedding of PTs among all samples; b, visualization of cell-cell junctions and transitions by RNA velocity with parameters of threshold =0.01; c, the proportion of unspliced/spliced mRNA among all samples and different IFTA groups; d, velocity length and confidence of PTs among different IFTA groups and Seurat clusters. Velocity length indicates cell differentiation pace. The darker red represents a faster pace. Velocity confidence indicates whether the differentiation direction is determined.

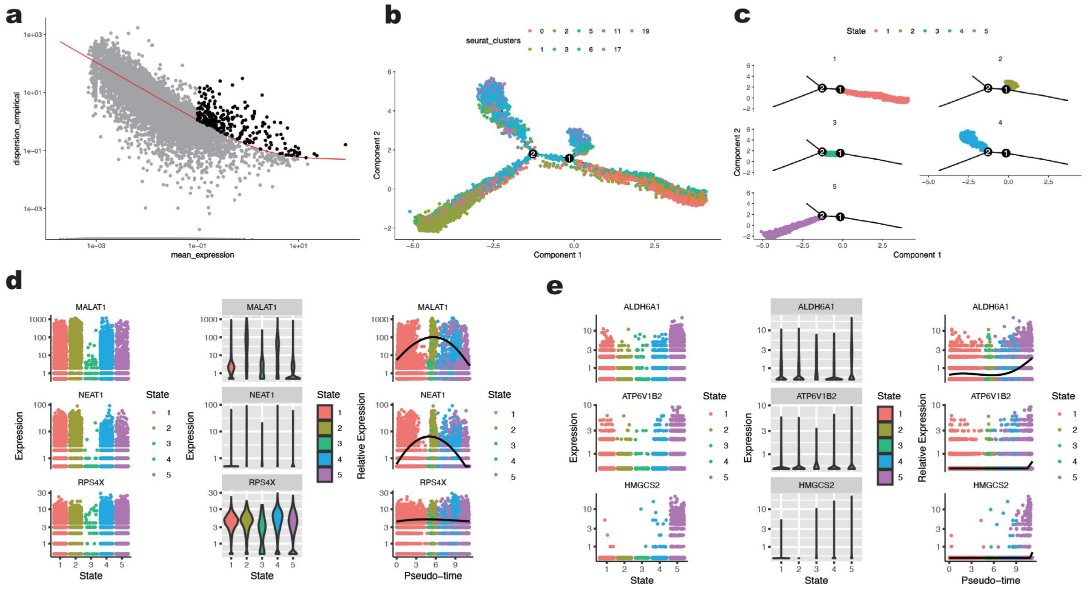

Supplementary Figure S18. Pseudotime trajectory analysis of PTs among different IFTA kidneys

a, DEGs with $p < 0.01$ between cells for further trajectory analysis were dot plotted in black; b-c, trajectory plot of Monocle2, to demonstrate the distribution of Seurat clusters (b) and cell state (c) along the pseudotime curve for IFTA development; d-e, representative expression changes of genes in Type 2 (d) and Type 3 (e) group (Figure 3g) by hierarchically clustering.

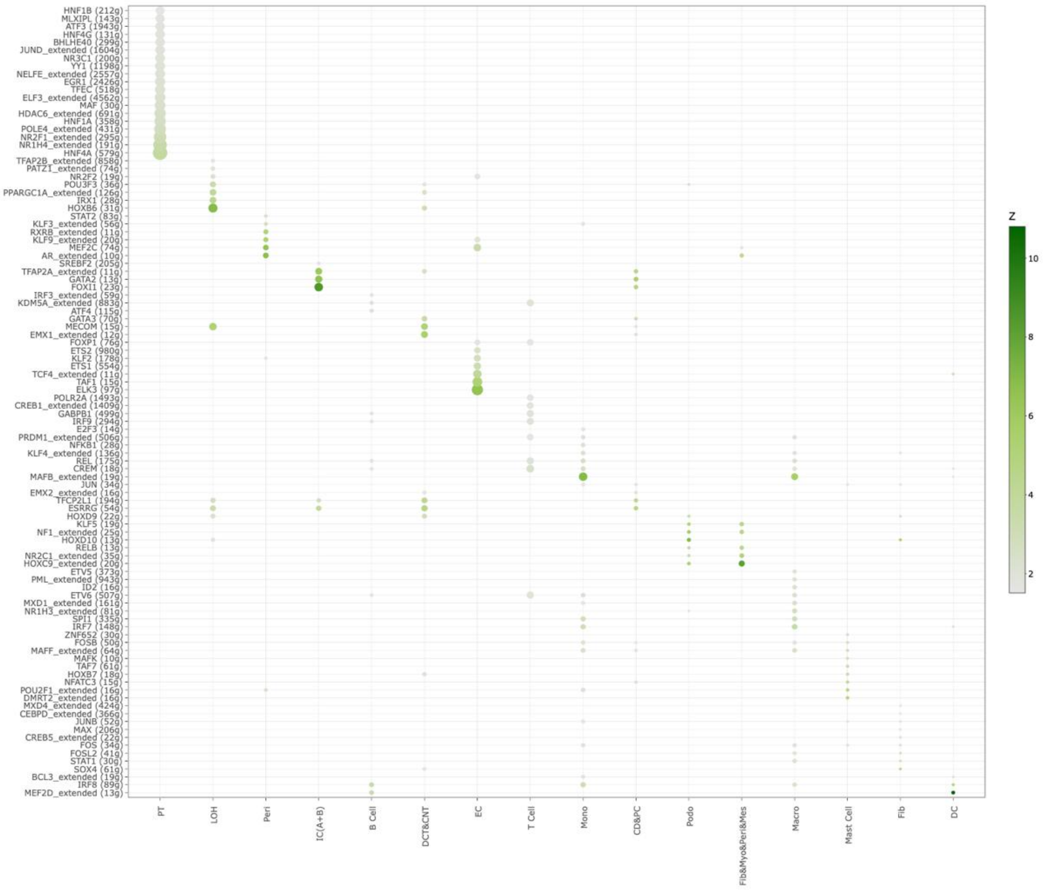

Supplementary Figure S19. Cell type specific regulons calculated by regulation AUC rssplot

Cell-type-specific transcriptional factors using RSS algorithm [26].

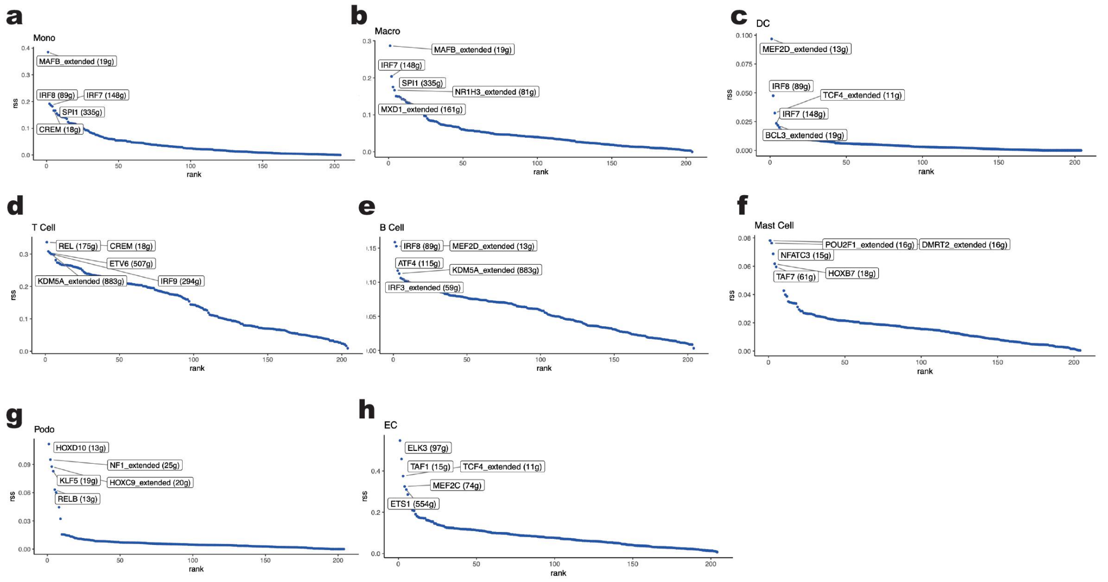

Supplementary Figure S20. Top 5 regulons in specific cell type

Regulon, transcription factors and corresponding candidate target gene group

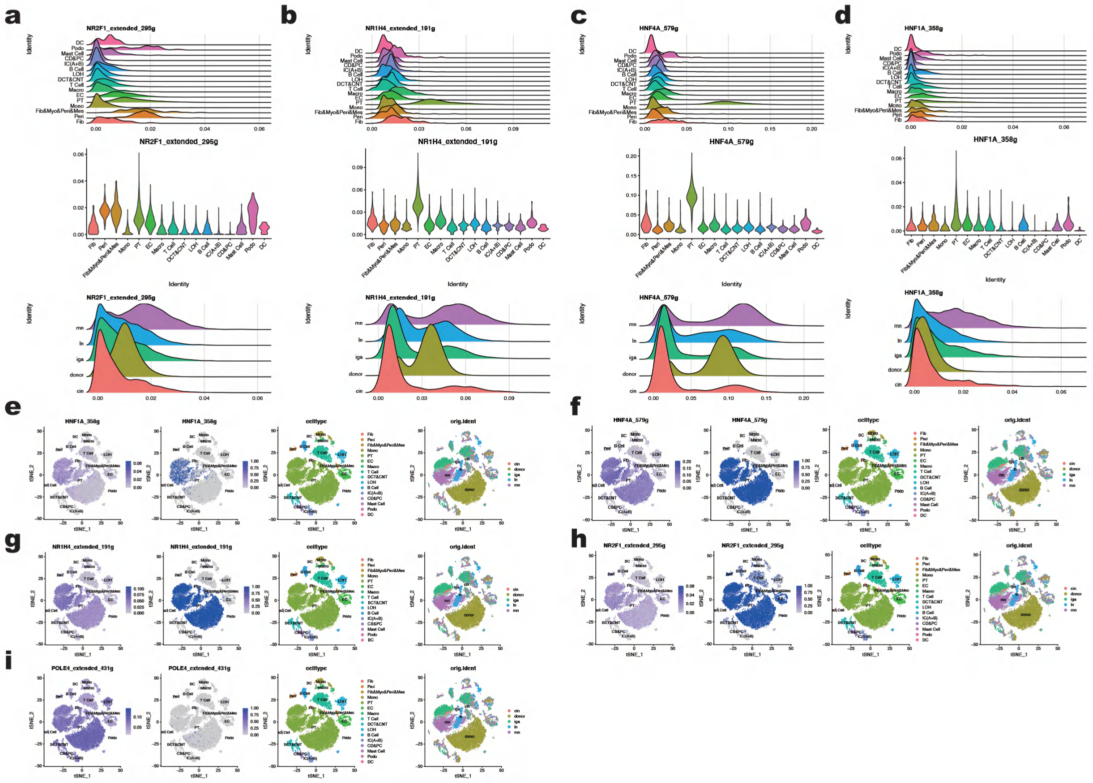

Supplementary Figure S21. The expression of the top regulons of proximal tubular epithelial cells (PT) a-d, ridgeplot (up), and violin plot (middle) of the expression of the top regulons (transcription factors and corresponding candidate target genes) of PTs in all cell types and ridgeplot (down) of the expression of the top regulons (transcription factors and corresponding candidate target genes) of PTs in all samples; e-i, featureplots of the expression and distribution of the top regulons of PTs by the average algorithm(left), binary algorithm(middle left) across cell types (middle right) and samples (right) using tSNE clustering.

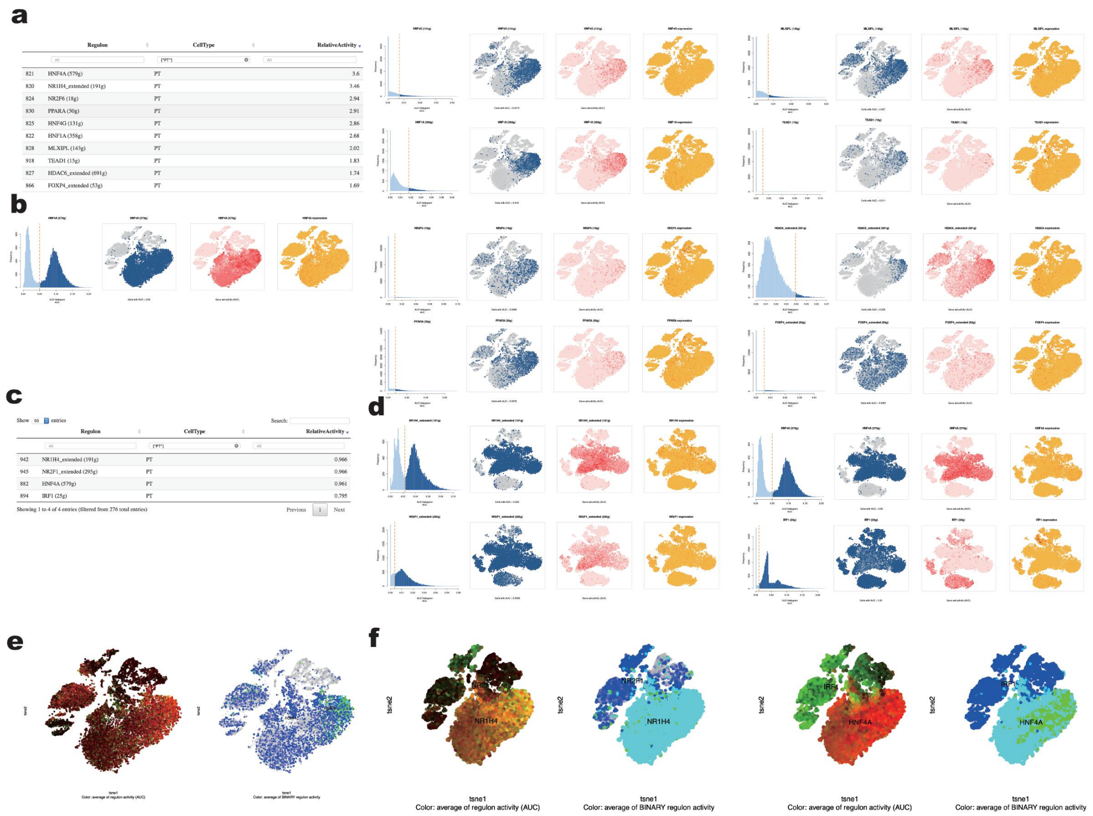

Supplementary Figure S22. The activity of the top regulons of PTs

a-b, the activity of the top regulons of proximal tubular epithelial cells (PT) by average algorithm; c-d, the activity of the top regulons of proximal tubular epithelial cells (PT) by binary algorithm; e, the activity of top regulons by average algorithm visualized by t-SNE screening by the threshold of (b); f, the activity of top regulons by binary algorithm visualized by t-SNE screening by the threshold of (d).

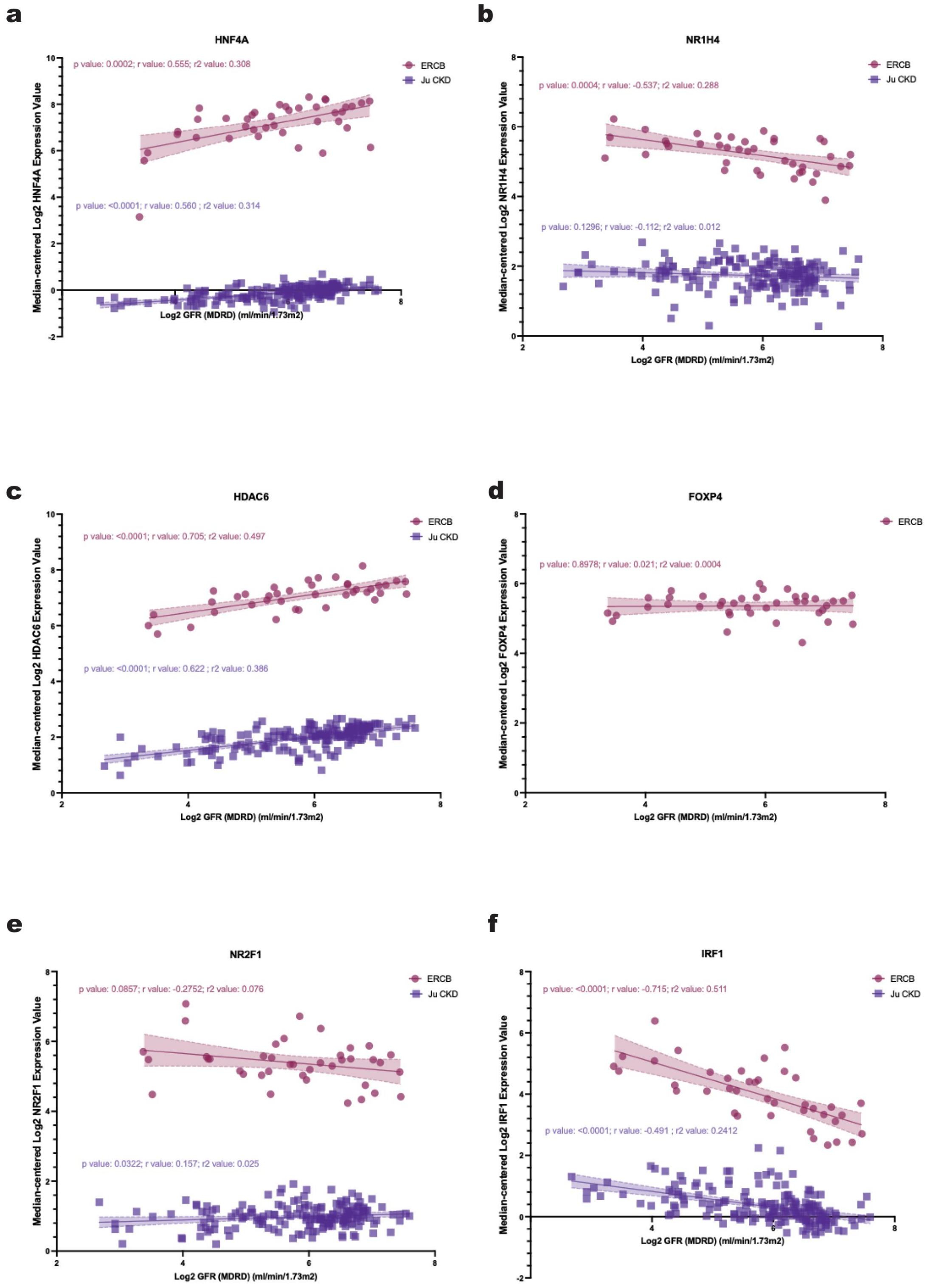

Supplementary Figure S23. Clinical importance of HNF4A, HDAC6, NR1H4, IRF1

Correlation analysis of HNF4A, NR1H4, HDAC6, FOXP4, NR2F1, IRF1 (a-f in order) with eGFR using the RNA-seq data of ERCB cohort[40] and microarray data of Ju CKD cohort[41] downloaded from Nephroseq website https://nephroseq.org/resource/login.html.

a

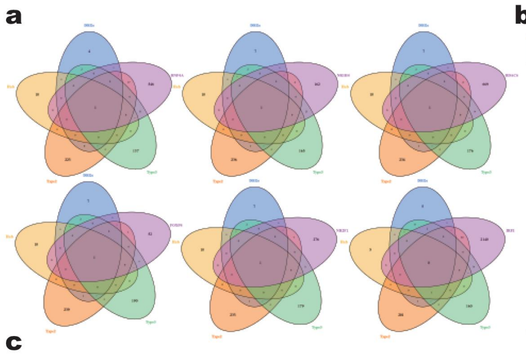

b

DEGs	chang	TF∩DEG	Best motif
NAT8	down	HNF4A	Not direct
STK19	down	HNF4A	Not direct
RARRES2	down	HNF4A	Not direct
CRYAB	down	/	
FABP1	down	/	
RPL34	up	IRF1	Not direct
MAP3K1	up	IRF1	Not direct
RPS14	up	IRF1	Not direct
SEMA5A	up	HNF4A	taipale_cyt_meth__HNF4A_NRGGTCAAAGTCCRN_eDBD_meth_repr
CCDC146	up	IRF1	Not direct

c

gene	gene_function	TF	highConf Annot	nMotifs	bestMotif	NES	motif Db	coex Module	spear Cor	Coex Weight
PAH	monocle2_type3 cluster	NR1H4	TRUE	1	stark__GTCANTNAAC	3.59	10kb	top50	0.232418	0.007209
ATF3	monocle2_type2 cluster	IRF1	TRUE	16	swissregulon__hs__IRF1_2_7.p3	4.76	10kb	top50	0.387943	0.019246
PSMB9	monocle2_type2 cluster	IRF1	TRUE	14	swissregulon__hs__IRF1_2_7.p3	4.76	10kb	top50	0.268921	0.010348
B2M	monocle2_type2 cluster	IRF1	TRUE	8	hocomoco__IRF1_MOUSE.H11MO.0.A	3.06	10kb	top1sd	0.374947	0.005961
MDK	monocle2_type2 cluster	IRF1	TRUE	9	hocomoco__IRF1_MOUSE.H11MO.0.A	3.06	10kb	top1sd	0.309662	0.004564
TNFSF10	monocle2_type2 cluster	IRF1	TRUE	7	hocomoco__IRF1_MOUSE.H11MO.0.A	3.06	10kb	top1sd	0.260688	0.005355

d

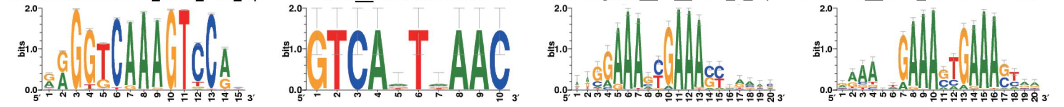

e

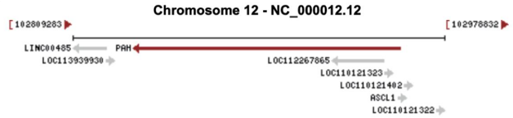

Matrix ID	Name	Score	Relative score	Sequence ID	Start	End	Strand	Predicted sequence
MA1110.1	MA1110.1.NR1H4	7.554371	0.8113270100298828	NC_000012.12:c102919244-102917244	312	322	+	TCATTGCCCTC
MA1110.1	MA1110.1.NR1H4	8.933066	0.8449352415443259	NC_000012.12:c102919244-102917244	1216	1226	-	GCAATGAACAA
MA1110.1	MA1110.1.NR1H4	8.702541	0.8393157713991501	NC_000012.12:c102919244-102917244	1001	1011	-	TTACTGACCAG

Supplementary Figure S24. Prediction of potential targets of HNF4A, HDAC6, NR1H4, IRF1

a, Venn diagram of the target genes of HNF4A, NR1H4, HDAC6, FOXP4, NR2F1, IRF1 from SCENIC analysis with the hub genes from the differential expression, WGCNA, Cellchat, and Monocle2 pseudotime (type2, type3) analysis above in PT heterogeneity; b-d, The most significantly enriched high-confidence binding motif of TFs and hub genes related to PT heterogeneity (b-c) and detailed sequence (d); e, prediction for the binding motif of NR1H4 on PAH using JASPER database. PAH location in Chromosome 12 (left) and potential binding motif on PAH (within 2000bp upstream of translation starting site of PAH transcript NM_000277.3); The most possible binding motif of NR1H4 on PAH might be TCATTGCCCTC 312 to 322bp upstream of translational start site of PAH which has been marked red.

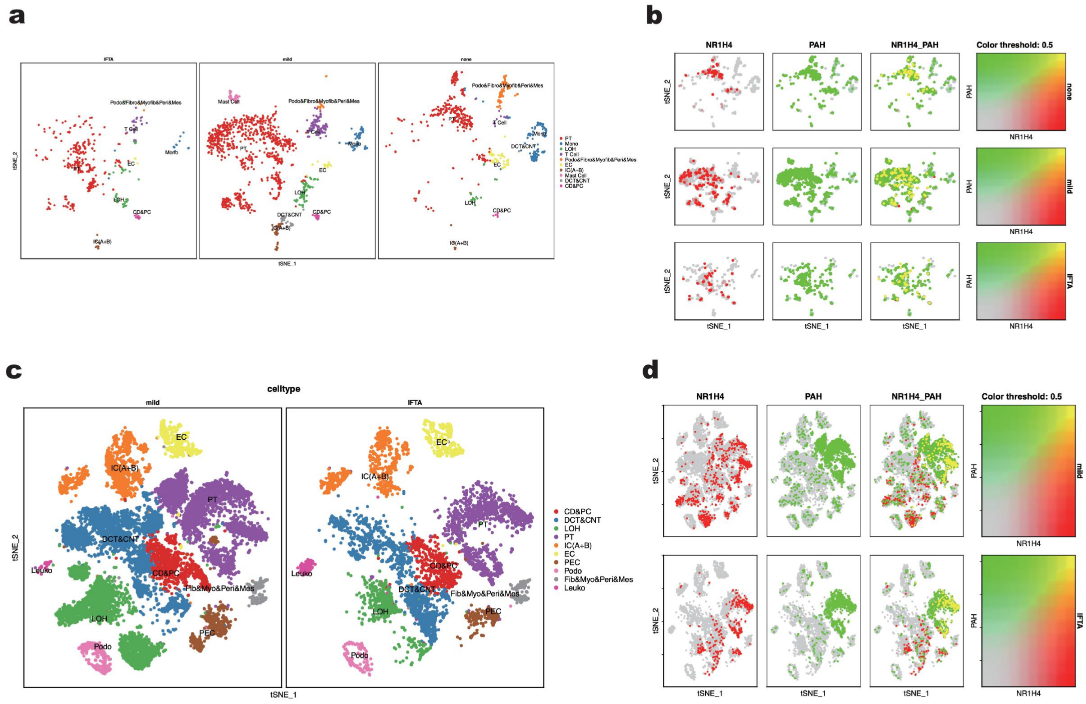

Supplementary Figure S25. Co-localization and expression of NR1H4 and PAH in public single cell sequencing data

a, unbiased clustering of cells from all kidney samples (GSE127136)[27] by t-SNE. Fewer PTs were observed in IFTA samples (Graded T1 by Oxford classification of IgA nephropathy) while more immunes cells in mild samples (Graded T0 by Oxford classification of IgA nephropathy). None samples contain 4 renal clear cell carcinoma,1 urothelial carcinoma, and 1 nephrapostasis, of which cell proportion might be different from healthy kidney due to tumor or nephrapostasis microenvironment. The top10 markers for each cell cluster and type were in Supplementary Table S10; b, co-localization and relative high expression of NR1H4 and PAH in PTs among samples with different grades of IFTA; c, unbiased clustering of cells from all samples (GSE131882)[28] by t-SNE. Fewer PTs and more leukocytes were observed in IFTA samples (11%-25% IFTA) compared to mild samples (1-10% IFTA). The top10 markers for each cell cluster and type were in Supplementary Table S11; b, co-localization and relatively high expression of NR1H4 and PAH in PTs among samples with different grades of IFTA;

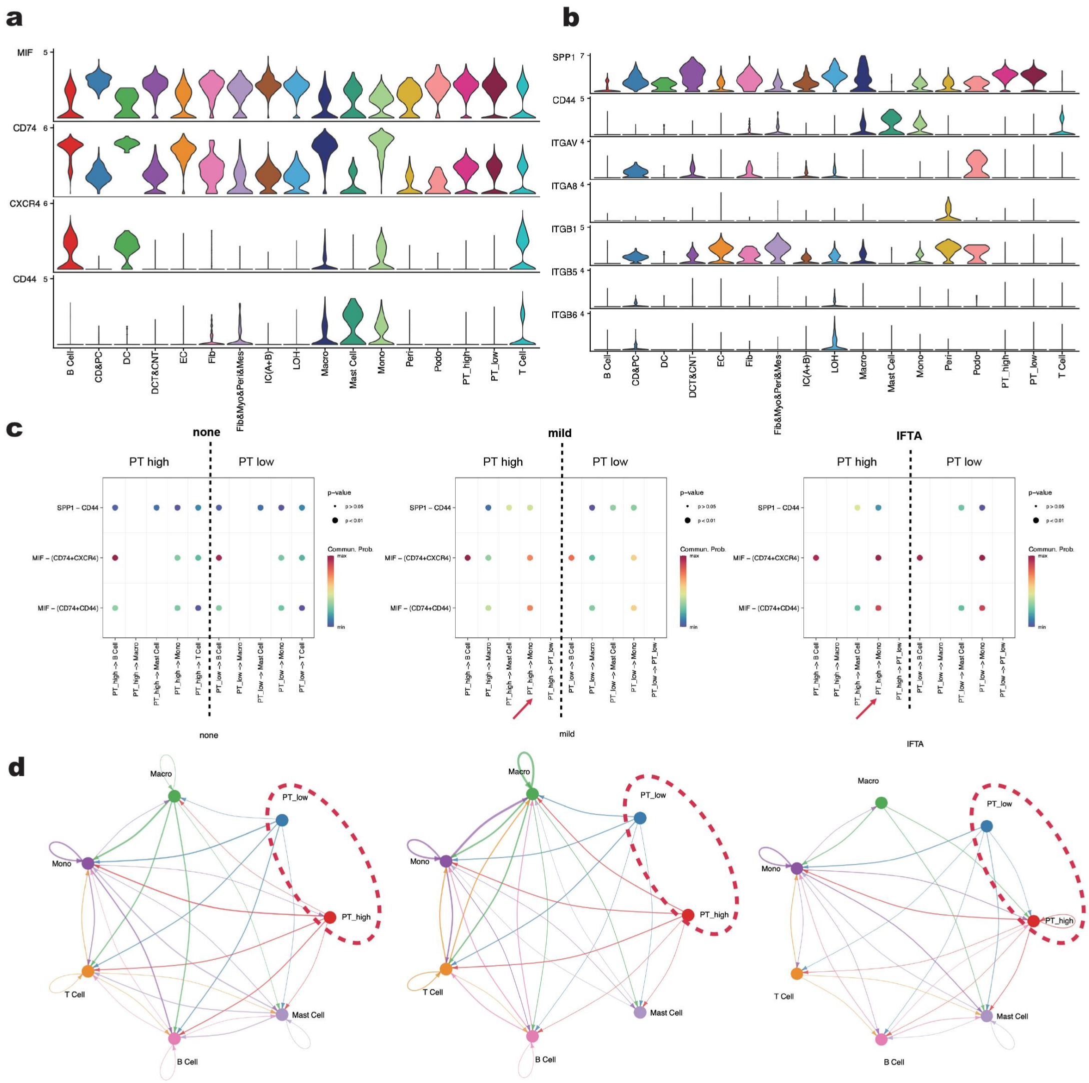

Supplementary Figure S26. Cell-to-cell contact difference between NR1H4 highly-expressed PTs and NR1H4 lowly-expressed PTs

a-b, violin plot of expression of MIF pathway(a) or SPP1 pathway(b) related genes across cell types of all samples; c, bubble plot of the main signaling pathway from PTs to infiltrated immune cells among different grades of IFTA; d, circle plot summarizing the interactions between PTs and infiltrated immune cells among different grades of IFTA. PT high, NR1H4 highly-expressed PTs (count of NR1H4 >0); PT low, NR1H4 lowly-expressed PTs (count of NR1H4 =0).

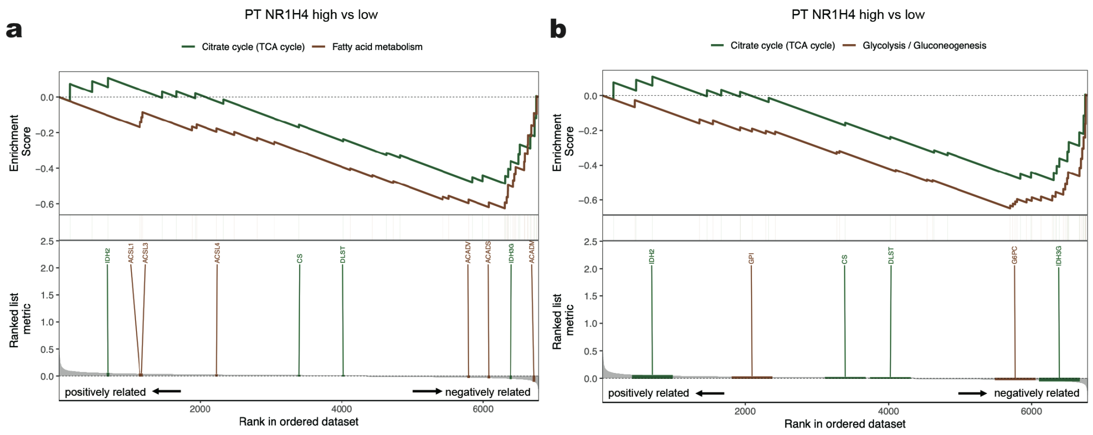

Supplementary Figure S27. Decease of TCA cycle, glycolysis, and fatty acid oxidation in NR1H4 highly-expressed PTs compared to NR1H4 lowly-expressed PTs

a-b, ssGSEA of DEGs of NR1H4 highly-expressed PTs (count of NR1H4 >0) versus NR1H4 lowly-expressed PTs (count of NR1H4 =0) by FindMarker function with parameters of min.pct setting at 0.01 and logfc.threshold at 0.01. The analysis was performed based on KEGG database. Detailed DEGs and enriched pathways were in Supplementary Table S12-13. (a), TCA cycle pathway (NES=-1.7908, adj.p=0.0078) and fatty acid metabolism pathway (NES=-2.5165, adj.p=0.0000). Interested genes encoding key enzymes of TCA cycle (CS, IDH3G, IDH2, OGDH, DLST, DLD), fatty acid activation (ACSS1-3, ACSL1, ACSL3-6, SLC27A2, ACSF2-3, ACSM2A-2B, ACSM1, ACSM3-6), fatty acid transport (CROT, CRAT, CPT1), fatty acid β-oxidation (ACAD family -S, -M, -L, -VL) were marked according to their correlation to the pathway if they were in the DEGs; (b), TCA cycle and glycolysis/gluconeogenesis (NES=-2.6609, adj.p=0.0000). Interested genes encoding key enzymes of TCA cycle (CS, IDH3G, IDH2, OGDH, DLST, DLD), or glycolysis (HK, GPI, G6PC, PKLR, PKM) were marked according to their correlation to the pathway if they were in the DEGs.

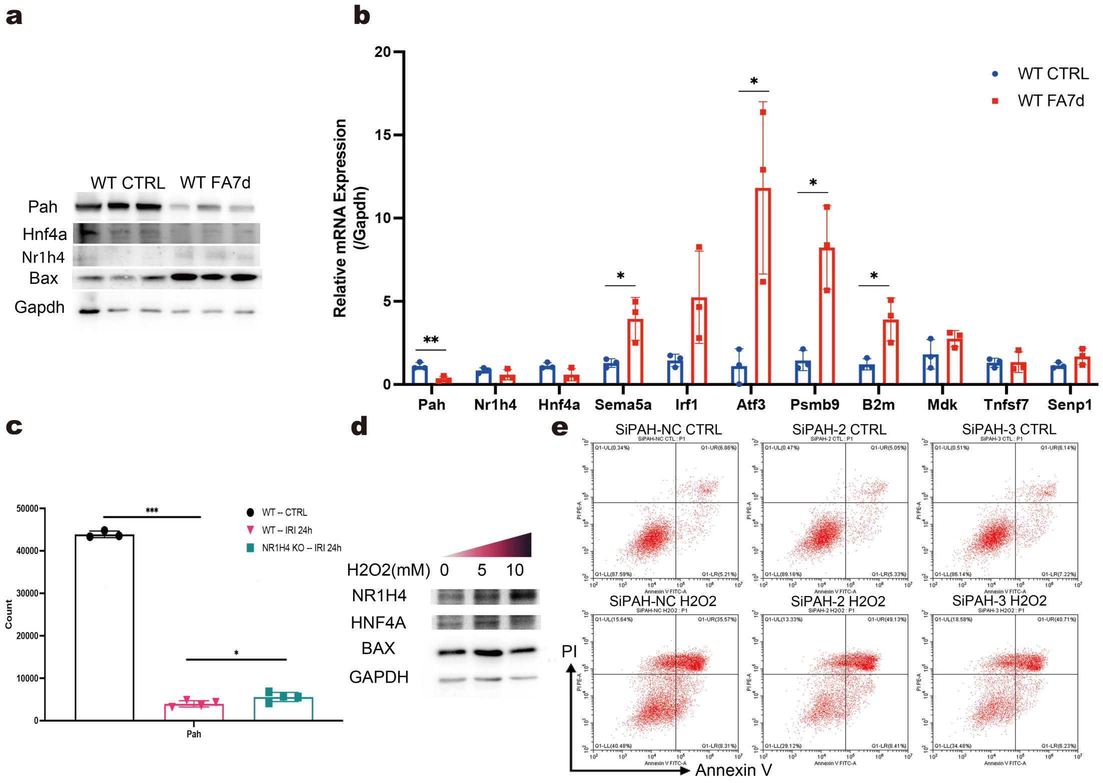

Supplementary Figure S28. In vivo and ex vivo validation of NR1H4-PAH

a-b, protein and mRNA expression of Nr1h4 and Pah in folic-acid induced kidney injury mice; c, expression of Nr1h4 and Pah in bulk RNA-seq data of WT and Nr1h4 -/- mice with or without ischemia-reperfusion induced acute kidney injury (IRI) from our previous study; d, expression of Nr1h4 and Pah in HK2 cells with or without H2O2 stimulation; e, flow cytometry analysis of apoptotic cells in HK-2 cells transfected with PAH siRNAs with or without H2O2 treatment by Annexin V-PI staining.

Supplementary Tables

Table S1. Clinical information of the participant

M=male; eGFR, estimated GFR(MDRD)=186 × (Scr/88.4)^(−1.154) × age^(−0.2030); N, No; Y, Yes; DM, diabetes; HTN, hypertension; IgAN, IgA Nephropathy; CIN, Chronic Tubulointerstitial Nephritis; MN, Membraneous Nephropathy; LN, Lupus Nephropathy; -, Normal; *, the IgAN patient lost follow-up at the third month, the laboratory results were from his last visit.

Table S2. Marker genes for each cell cluster (attached file)

Table S3. DEGs of each cell clusters from IFTA versus none to mild IFTA group

Table S4. DEGs of PTs from IFTA versus none to mild IFTA group

Table S5. Enriched pathway based on DEGs of PTs from IFTA versus none to mild IFTA group

KEGG database was used for enrichment analysis.

Table S6. Gene names of the four expression-pattern clusters in trajectory analysis of PT (attached file)

Table S7. Regulon and Target information (attached file)

Table S8. Enrichment of Target motif information (attached file)

Table S9. The enriched best motif of high confidence of HNF4A, NR1H4, IRF1 (attached file)

Table S10. Top 10 marker genes for each cell cluster of GSE127136 (attached file)

Table S11. Top 10 marker genes for each cell cluster of GSE131882 (attached file)

Table S12. DEGs of NR1H4 highly-expressed PTs versus NR1H4 lowly-expressed PTs

Table S13. Enriched pathway based on DEGs of PTs from IFTA versus none to mild IFTA group

KEGG database was used for enrichment analysis.
